# ﻿ *Onygenales* from marine sediments: diversity, novel taxa, global distribution, and adaptability to the marine environment

**DOI:** 10.3897/imafungus.16.158470

**Published:** 2025-09-04

**Authors:** Daniel Guerra-Mateo, Josepa Gené, Pierre Becker, José F. Cano-Lira

**Affiliations:** 1 Universitat Rovira i Virgili, Facultat de Medicina i Ciències de la Salut, Unitat de Micologia i Microbiologia Ambiental, Reus, Spain Universitat Rovira i Virgili Reus Spain; 2 Institut Universitari de Recerca en Sostenibilitat, Canvi Climàtic i Transició Energètica (IU-RESCAT), Vila-seca, Spain Institut Universitari de Recerca en Sostenibilitat, Canvi Climàtic i Transició Energètica Vila-seca Spain; 3 BCCM/IHEM Fungi Collection: Human & Animal Health, Mycology and Aerobiology, Sciensano, Brussels, Belgium BCCM/IHEM Fungi Collection: Human & Animal Health, Mycology and Aerobiology Brussels Belgium

**Keywords:** Cellulolytic, chitinolytic, fungal biogeography, halotolerance, keratinolytic, marine sediments, phylogeny

## Abstract

The *Onygenales* represent a versatile group of fungi that primarily inhabit soils, degrading cellulose and/or keratin. While some are known human pathogens, others are osmotolerant or colonize chitin substrates such as insects. The marine environment, characterized by 3.5% salinity and chitin as the dominant polysaccharide, represents an intriguing niche for these fungi. However, fungal diversity in this environment remains poorly studied. This study investigated the culturable diversity of *Onygenales* in marine sediments, explored their global biogeography, and assessed their adaptability to marine conditions. Marine sediments were collected near river mouths and other coastal areas along the Catalan coast (Spain). Identification was based on a polyphasic approach; global distribution patterns were assessed through the GlobalFungi database, and adaptability was evaluated through osmotolerance and substrate degradation assays (cellulose, chitin, keratin). We recovered 32 strains, of which 24 represented 16 known species distributed in *Gymnascella*, *Gymnoascus*, *Narasimhella*, and *Sporendonema (Gymnoascaceae)*; *Malbranchea (Malbrancheaceae)*; *Myriodontium (Neoarthropsidaceae)*; and *Aphanoascus* and *Byssoonygena (Onygenaceae)*. The remaining eight strains were delineated as six novel species, including a new genus: *Gymnoascoideus alboluteus***sp. nov.**, *Malbranchea parafilamentosa***sp. nov.**, *M. sedimenticola***sp. nov.**, *M. seminuda***sp. nov.**, *M. sexualis***sp. nov.**, and *Deilomyces minimus***gen. et sp. nov.** In addition, all strains degraded cellulose, and most tolerated up to 10% NaCl. Only four species that also degraded chitin (*Malbranchea parafilamentosa*, *M. sexualis*, *Myriodontium keratinophilum*, and *Sporendonema casei*) could be considered facultative marine fungi. This work evidences the great diversity of onygenalean fungi in marine sediments and underscores their metabolic adaptability to marine conditions.

## ﻿Introduction

The order *Onygenales* comprises a versatile group of ascomycetous fungi. Although they are known for their pathogenic behavior in animals and humans, their natural habitat is soil and dung. In particular, these fungi inhabit substrates such as agricultural soil and both herbivore and carnivore dung, where they use compounds like cellulose and/or keratin as their primary source of carbon for nutrition (Kandemir et al. 2022). The family *Spiromastigoidaceae* is characterized by its ability to degrade cellulose, the most abundant polysaccharide in terrestrial environments (Hirooka et al. 2016; Kandemir et al. 2022). In contrast, families such as *Arthrodermataceae*, *Ajellomycetaceae*, and *Onygenaceae* have lost the ability to degrade cellulose, instead specializing in keratin degradation (Muñoz et al. 2018). These fungi colonize keratin-rich, animal-associated substrates such as horns, feathers, hooves, and hair, which are often found in carnivore dung, but also exhibit pathogenic behavior in both humans and animals (Chaturvedi and de Hoog 2020). In addition, other families have explored distinct ecological adaptations, such as osmotolerance, which has been a key evolutionary driver enabling the colonization of substrates in extreme environments like caves and desert soils (Zhou et al. 2016; Zhang et al. 2017). The families *Ascosphaeraceae* and *Gymnoascaceae* are characterized by their tolerance to osmotic stress. Members of *Gymnoascaceae* are often associated with habitats with high salt concentrations. In this family, the genus *Sporendonema* is particularly notable for its association with cheese and dried meat (Scaramuzza et al. 2015; Kandemir et al. 2024). On the other hand, members of *Ascosphaeraceae* are predominantly recovered from bees and beehives, where they tolerate high sugar concentrations and can colonize bee larvae through the ingestion of ascospores (Aronstein and Murray 2010). Although there is no evidence of chitin degradation during larvae colonization, onygenalean fungi are known to produce various chitinases, suggesting chitinolytic capabilities in different ecological contexts (Goughenour et al. 2021). In fact, members of the family *Neogymnomycetaceae* and the species *Gymnoascus exasperatus* have been described from bat guano, a chitin-rich substrate (Nováková and Kolařík 2010; Kaya et al. 2014; Zhang et al. 2017; Kandemir et al. 2022).

In this context, aquatic environments seem to be suitable habitats for the presence of onygenalean fungi. These habitats are characterized by chitin as the dominant polysaccharide (Souza et al. 2011). Freshwater sediments harbor a diverse community of onygenalean fungi, including species common in terrestrial habitats, as well as clinically relevant fungi and novel taxa (Marin-Felix et al. 2015; Torres-Garcia et al. 2023). However, little is known about their presence in marine environments, where fungi must tolerate high salinity (3.5% NaCl), high hydrostatic pressure, and limited availability of carbon and nitrogen sources (Souza et al. 2011; Kanakidou et al. 2020). In these habitats, chitin represents the primary source of these molecules, with bacteria playing a major role in its degradation, often at a rate comparable to its production (Howard et al. 2003). There are three forms of chitin in nature (α, β, and γ), with α-chitin being the most abundant. It is found in fungal cell walls, insect cuticles, and the exoskeletons of crabs and shrimps (Souza et al. 2011). The role of fungi in marine nutrient cycles remains largely unclear. Nevertheless, onygenalean fungi have been isolated from marine sediments, seagrasses, and invertebrate marine animals, providing evidence of their metabolic activity in this environment (Garzoli et al. 2015; Marchese et al. 2020; Poli et al. 2020; Guerra-Mateo et al. 2024).

Detecting onygenalean fungi in marine environments using standard culture media is often unreliable. However, metabarcoding analyses have identified members of the families *Ajellomycetaceae* and *Onygenaceae* (Li et al. 2016; Picard 2017; Barone et al. 2018). Culture-dependent surveys generally recover only a few onygenalean species, alongside more abundant representatives from other fungal orders such as *Eurotiales*, *Pleosporales*, and *Hypocreales* (Landy and Jones 2006; Jones et al. 2019; Marchese et al. 2021; Guerra-Mateo et al. 2024). Techniques specifically designed to target onygenalean fungi, such as hair-baiting or supplementing culture media with cycloheximide, have proven effective for their detection in coastal sediments (Ulfig et al. 1997, 1998). In particular, the use of cycloheximide has been successful in recovering *Onygenales* in culture, taking advantage of their resistance to this protein synthesis inhibitor (Torres-Garcia et al. 2023). More recently, skimmed milk flocculation (SMF) has been described as an effective method for isolating culturable fungi from marine sediments (Guerra-Mateo et al. 2024). The combination of cycloheximide and SMF could represent a promising strategy for improving the recovery of *Onygenales* from marine environments.

This study aimed to investigate the diversity of culturable onygenalean fungi associated with marine sediments from the western Mediterranean Sea basin, a recognized hotspot for fungal diversity (Coll et al. 2010; Guerra-Mateo et al. 2024). To achieve this, we applied a dual approach that combined direct plating of the sediment samples with an SMF pretreatment, using four different culture media, including one supplemented with cycloheximide. We provide a detailed characterization of the culturable community of *Onygenales*, delineate the taxonomy of several noteworthy strains under a consolidated species concept, and introduce a new genus, a new species of *Gymnoascoideus*, and four new species of *Malbranchea*. Additionally, since most of the identified species are based on an isolated singleton (Cazabonne et al. 2024), we examined their global distribution and ecology using the GlobalFungi database (https://globalfungi.com) and evaluated their potential roles and adaptability to marine sediments through a series of physiological tests.

## ﻿Methodology

### ﻿Study area and sampling

**Figure 1.**
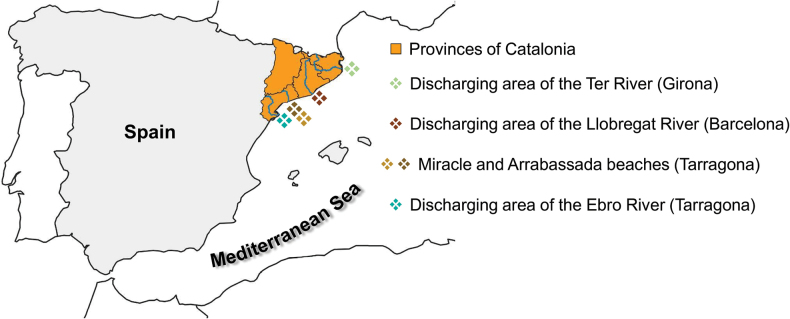
Collection sites throughout the coasts of Catalonia, Spain, in the western Mediterranean Sea. The map was created using QGIS (v3.34.5; QGIS Development Team 2024).

Marine sediments examined in this study were collected during several surveys conducted along the coast of Catalonia (Spain) between 2021 and 2023. Sampling sites included the area in front of two beaches on the Tarragona coast (Miracle and Arrabassada), located near the discharge area of the Francolí River, and the discharge areas of the rivers Ebro (Tarragona Province), Ter (Girona Province), and Llobregat (Barcelona Province) (Fig. 1). The latter three locations are included in the natural parks of the Ebro Delta, the Medas Islands, and the Llobregat Delta, respectively.

At each site, samples were collected in sets of two independent surveys, and sediments were recovered from around 6, 13, 20, and 27 m depth. The Llobregat River site was an exception, with only one survey conducted, during which samples were collected from 20 and 30 m depths. At each depth, four sub-samples were taken from 15 cm below the seabed surface, using 50 mL plastic tubes. Throughout the collection process, tubes were stored in a refrigerated container until their arrival at the laboratory.

### ﻿Culturing and fungal isolation

At each area, sediment samples from different depths were treated separately. However, the sub-samples from each depth were mixed in a container, vigorously shaken, and poured onto a plastic tray with several layers of sterile filter paper to remove excess water. Subsequently, each mixed sample was assessed through both direct plating and an SMF pre-treatment, following the procedure explained in Guerra-Mateo et al. (2024).

The culture media used for isolation were dichloran rose bengal chloramphenicol agar (DRBC; 5 g peptone, 10 g glucose, 1 g KH_2_PO_4_, 0.5 g MgSO_4_, 25 mg rose bengal, 200 mg chloramphenicol, 2 mg dichloran, 15 g agar, 1 L distilled water); potato dextrose agar (PDA; Condalab, Madrid, Spain) supplemented with 2 g/L of cycloheximide (PDA+C); and two additional media made with seawater, 3% malt extract agar (SWMEA3%; 30 g malt extract, 5 g mycological peptone, 15 g agar, 1 L seawater) and oatmeal agar (SWOA; 30 g oatmeal, 15 g agar, 1 L seawater). To inhibit bacterial growth, 5 mL of chloramphenicol (15 g/L ethanol) was added to SWMEA3%, SWOA, and PDA+C media.

For direct plating, 1 g of sediment from each sample was distributed across two Petri dishes per medium and mixed with either melted PDA+C, SWMEA3%, or SWOA at 45 °C. In the case of DRBC, only 0.5 g of sediment was distributed across two Petri dishes to address the fast-growing fungi. A similar methodology was applied to the sediment pre-treated with SMF, with the exception that 1 mL of floccule from each sample was distributed across two Petri dishes for PDA+C, SWMEA3%, and SWOA, and 0.5 mL of floccule was mixed with DRBC. Each sample was cultured in duplicate for both approaches; one set of plates with different media was incubated at 22–24 °C, and the other at 15 °C. All cultures were incubated in darkness and examined weekly under a dissecting microscope for up to 5–8 weeks.

Pure cultures were obtained on PDA from fragments of the colonies or conidia in the primary plates using a sterile dissection needle. These cultures were used to provide a preliminary morphological identification. Strains that did not exhibit sporulation were further subcultured to potato carrot agar (PCA; 20 g potato, 20 g carrot, 15 g agar, 1 L distilled water) or oatmeal agar (OA; 30 g oatmeal, 15 g agar, 1 L distilled water) to promote sporulation.

The strains obtained were morphologically examined for a presumptive generic identification. A maximum of three identical strains per sample were selected for sequencing based on the conserved morphological traits frequently displayed between phylogenetically close ascomycetous species (Gannibal 2022). The strains included in this study represented a selection of the onygenalean fungi recovered from the marine sediment samples (Table 1). Identification of the strains at the species level was based on a polyphasic approach under the consolidated species concept, combining multi-locus phylogenetic analyses, morphology, and ecological data (Quaedvlieg et al. 2014). Metadata about the identified strains has been deposited in the Global Biodiversity Information Facility (GBIF, https://doi.org/10.15468/emtvnj, accessed in July 2025).

**Table 1.** Linked data table of collection details from the strains studied in the present work and GenBank accession numbers of the barcodes used for identification.

Taxon name	Strain^1^	Source	Locality	Depth (m)	Medium	Technique	Temp.^2^ (°C)	GenBank Accessions^3^				Reference
								ITS	LSU	*tub*2	*rpb*2	
*Aphanoascus crassitunicatus*	FMR 20176	Marine sediment	Arrabassada	13	PDA+C	Direct Culture	15	PP273949				Guerra-Mateo et al. 2024
*Aphanoascus fulvescens*	FMR 20116	Marine sediment	Arrabassada	27	SWMEA3%	Direct Culture	15	PP273928				Guerra-Mateo et al. 2024
	FMR 20522	Marine sediment	Ebro Delta	27	SWMEA3%	Flocculation	25	**PV474127**				
*Byssoonygena ceratinophila*	FMR 19558	Marine sediment	Miracle	13	DRBC	Direct Culture	15	PP273870	PP342588			Guerra-Mateo et al. 2024
*Deilomyces minimus*	FMR 20744^T^	Marine sediment	Ter	27	PDA+C	Flocculation	25	**PQ859450**	**PQ849171**			
*Gymnascella dankaliensis*	FMR 20162	Marine sediment	Arrabassada	27	PDA+C	Direct Culture	25	PP273939				Guerra-Mateo et al. 2024
	FMR 19625	Marine sediment	Miracle	27	PDA+C	Direct Culture	15	PP273888				Guerra-Mateo et al. 2024
	FMR 19616	Marine sediment	Miracle	20	SWMEA3%	Direct Culture	25	PP273883	PP342594			Guerra-Mateo et al. 2024
*Gymnascella nodulosa*	FMR 20375	Marine sediment	Ebro Delta	15	PDA+C	Direct Culture	25	**PQ859451**				
*Gymnascella udagawae*	FMR 20467	Marine sediment	Ebro Delta	27	SWOA	Direct Culture	25	**PQ859452**				
	FMR 20506	Marine sediment	Ebro Delta	27	PDA+C	Direct Culture	15	**PQ859453**				
	FMR 20769	Marine sediment	Ebro Delta	27	DRBC	Direct Culture	15	**PQ859454**				
*Gymnoascoideus alboluteus*	FMR 19992^T^	Marine sediment	Arrabassada	27	PDA+C	Direct Culture	15	PP273911	**PV474129**			Guerra-Mateo et al. 2024
*Gymnoascus exasperatus*	FMR 20452	Marine sediment	Ebro Delta	27	PDA+C	Direct Culture	25	**PQ859455**	**PQ849172**	**PQ891866**		
*Gymnoascus reessii*	FMR 19395	Marine sediment	Miracle	27	SWMEA3%	Direct Culture	25	PP273850				Guerra-Mateo et al. 2024
*Gymnoascus uncinatus*	FMR 21364	Marine sediment	Llobregat	30	PDA+C	Flocculation	15	**PQ859456**	**PQ849173**	**PQ891867**		
*Malbranchea irregularis*	CBS 149937 ^T^	Riparian sediment						ON720191	ON720730	OP425710	OP425719	Torres-Garcia et al. 2023
*Malbranchea longispora*	CBS 135817 ^T^	Soil						NR_164514	HG326874	**PQ891864**	**PV498619**	Rodriguez-Andrade et al. 2021
*Malbranchea multiseptata*	CBS 146931 ^T^	Human, bronchial washing						NR_178131	NG_088053	**PQ891865**		Rodriguez-Andrade et al. 2021
*Malbranchea ostraviensis*	FMR 20173	Marine sediment	Arrabassada	27	DRBC	Flocculation	15	PP273946				Guerra-Mateo et al. 2024
*Malbranchea sinuata*	CBS 149938 ^T^	Riparian sediment						ON720195	ON720734	OP425704	OP425714	Torres-Garcia et al. 2023
*Malbranchea sedimenticola* (= *Malbranchea* sp. 1)	FMR 19564^T^	Marine sediment	Miracle	27	DRBC	Flocculation	15	PP344596	**PQ849174**	**PQ891868**	**PV498620**	Guerra-Mateo et al. 2024
	FMR 20150	Marine sediment	Arrabassada	27	PDA+C	Direct Culture	15	**PP344597**	**PQ849175**	**PQ891869**		
	FMR 21121	Marine sediment	Llobregat	19	PDA+C	Flocculation	15	**PQ859457**	**PQ849176**	**PV498618**	**PV498621**	
	CBS 319.61	Soil						MH858065	MH869635	**PQ891870**	**PV498622**	Vu et al. 2019
*Malbranchea seminuda* (= *Malbranchea* sp. 2)	FMR 19403^T^	Marine sediment	Miracle	27	SWMEA3%	Flocculation	15	PP344598	**PQ849177**	**PQ891871**		Guerra-Mateo et al. 2024
*Malbranchea parafilamentosa* (= *Malbranchea* sp. 3)	FMR 20151^T^	Marine sediment	Arrabassada	27	PDA+C	Direct Culture	25	PP344599	**PQ849178**	**PQ891872**	**PV498623**	Guerra-Mateo et al. 2024
	IHEM 28255	Bat fur						OU989280	OU641135	**PQ891873**	**PV498624**	
*Malbranchea sexualis* (= *Malbranchea* sp. 4)	FMR 20852^T^	Marine sediment	Ebro Delta	27	PDA+C	Direct Culture	15	**PQ859458**	**PQ849179**	**PQ891874**		
*Malbranchea zuffiana*	FMR 20086	Marine sediment	Miracle	20	PDA+C	Flocculation	15	PP273922	PP342596			Guerra-Mateo et al. 2024
*Myriodontium keratinophilum*	FMR 20839	Marine sediment	Ter	27	PDA+C	Direct Culture	25	**PV474128**	**PV474130**			
*Narasimhella hyalinospora*	FMR 20283	Marine sediment	Arrabassada	20	DRBC	Flocculation	15	PP273953				Guerra-Mateo et al. 2024
*Narasimhella poonensis*	FMR 19978	Marine sediment	Arrabassada	27	PDA+C	Direct Culture	25	PP273902				Guerra-Mateo et al. 2024
	FMR 19613	Marine sediment	Miracle	27	SWMEA3%	Flocculation	15	PP273882	PP342598			Guerra-Mateo et al. 2024
	FMR 19621	Marine sediment	Miracle	27	SWMEA3%	Flocculation	15	PP273886	PP342599			Guerra-Mateo et al. 2024
	FMR 19620	Marine sediment	Miracle	27	SWMEA3%	Direct Culture	25	PP273885	PP342597			Guerra-Mateo et al. 2024
*Sporendonema casei*	FMR 21361	Marine sediment	Llobregat	30	SWMEA3%	Flocculation	15	**PQ859460**				

^1^FMR: Facultat de Medicina i Ciències de la Salut, Reus, Spain. CBS: Culture collection of the Westerdijk Fungal Biodiversity Institute, Utrecht, The Netherlands. IHEM: BCCM/IHEM fungi collection, Brussels, Belgium. ^2^Temp. indicates temperature. ^3^Accession numbers in bold have been generated in this study. ^T^ indicates type strains.

### ﻿DNA extraction, PCR amplification, and sequencing

Genomic DNA was extracted using a chloroform/isopropanol protocol (Tripathy and Maharana 2017) and quantified with a Nanodrop 2000 (Thermo Scientific, Madrid, Spain). We amplified the ITS region and the LSU D1–D3 domain of the nrDNA using the primer pair ITS5/LR5 (Vilgalys and Hester 1990; White et al. 1990). These two regions were used as the main barcodes for taxonomic evaluation and identification. We also amplified the β-tubulin (*tub2*) and the second largest subunit of RNA polymerase II (*rpb2*) using the primer pairs Bt2a/Bt2b (Glass and Donaldson 1995) and RPB2-5F/RPB2-7R (Liu et al. 1999), respectively. The *tub2* region was used to resolve the phylogeny of closely related species in *Malbranchea* and *Gymnoascus*, and the *rpb2* region was only used to compare sister taxa in *Malbranchea* due to the limited availability of this region in public databases. Briefly, PCR conditions for ITS, LSU, *tub2*, and *rpb2* consisted of an initial denaturation step at 95 °C for 5 min, followed by 35 cycles of 30 s at 95 °C, 1 min at 56 °C, and 1 min at 72 °C, with a final extension step at 72 °C for 10 min. PCR products were purified and sequenced at Macrogen Corp. Europe (Madrid, Spain) using the same primers employed for amplification. Consensus sequences were assembled using SeqMan v. 7.0.0 (DNAStar Lasergene, Madison, USA) and can be found in Table 1.

The resulting sequences were compared with those available at the National Center for Biotechnology Information (NCBI), using the Basic Local Alignment Search Tool (BLAST; https://blast.ncbi.nlm.nih.gov/Blast.cgi, accessed in January 2025). A maximum similarity level of > 98% with ≥ 90% sequence coverage was used to determine related GenBank sequences. Accession numbers were carefully examined to identify those corresponding to type strains of accepted species, and these were combined with data from the most recent phylogenetic studies to perform phylogenetic analyses (Kandemir et al. 2022; Torres-Garcia et al. 2023). The accession numbers of the onygenalean strains included in the phylogenetic analyses are listed in the Suppl. material 1. The sequence alignments generated in this study have been deposited in Zenodo (https://doi.org/10.5281/zenodo.15373205, accessed in May 2025).

### ﻿Phylogenetic analyses

Sequence alignments were made using the ClustalW algorithm (Thompson et al. 1994) in MEGA (Molecular Evolutionary Genetics Analysis) software v. 6.0 (Tamura et al. 2013) and refined with MUSCLE (Edgar 2004) or adjusted manually as needed. Each genomic DNA region was aligned individually before being combined into a single dataset. The phylogenetic concordance among individual trees was visually assessed to identify incongruent results among clades with high statistical support. Following confirmation of concordance, the separate alignments were concatenated into a single data matrix in MEGA (Tamura et al. 2013).

Maximum likelihood (ML) analyses were conducted using IQ-TREE v. 2.3.6 (Nguyen et al. 2015; Minh et al. 2020) with ultrafast bootstrapping for the estimation of branch support (Minh et al. 2013; Hoang et al. 2018). The most suitable evolutionary model for each partition was estimated using ModelFinder (Chernomor et al. 2016; Kalyaanamoorthy et al. 2017), implemented in IQ-TREE, following the Bayesian information criterion. A bootstrap value ≥ 95 was considered significant (Minh et al. 2013; Hoang et al. 2018). Bayesian inference (BI) analyses were run using MrBayes v. 3.2.6 (Ronquist et al. 2012). The best substitution model for each locus was estimated using jModelTest v. 2.1.3, following the Akaike criterion (Guindon and Gascuel 2003; Darriba et al. 2012). Markov chain Monte Carlo (MCMC) sampling was performed for 10 million generations using four simultaneous chains (one cold chain and three heated chains), starting from a random tree topology. Trees were sampled every 1000^th^ generation or until the run was stopped automatically when the average standard deviation of split frequencies fell below 0.01. The first 25% of the trees were discarded as the burn-in phase of each analysis, and the remaining trees were used to calculate posterior probabilities (pp), representing branch support for the consensus trees. A pp value ≥ 0.95 was considered significant (Hespanhol et al. 2019). The resulting trees were plotted using FigTree v. 1.3.1 (http://tree.bio.ed.ac.uk/software/figtree/, accessed in March 2025).

### ﻿Species delimitation analyses in *Malbranchea*

*Malbranchea* strains were characterized through species delimitation analyses. We performed a coalescent-based method, Bayesian Poisson Tree Processes, bPTP (Zhang et al. 2013), and a distance-based method, Assemble Species by Automatic Partitioning, ASAP (Puillandre et al. 2021). These analyses were performed on the concatenated ITS–LSU–*tub2* alignment and the ITS single alignment. The input trees for the PTP analyses were inferred in IQ-TREE, as explained above. The analyses were conducted on the web server for bPTP (https://species.h-its.org/ptp/, accessed during November 2024) with 500,000 MCMC generations, thinning set to 100, burn-in of 10%, and a seed value of 123456. Convergence of the MCMC iterations was assessed by visualizing the log-likelihood trace plot, where the chain’s predominant exploration of high-likelihood locations was used as an indicator of convergence. The ASAP analyses were also performed for the concatenated and single ITS alignments using the matrix of pairwise nucleotide distances. This matrix was computed in MEGA v. 6.0 using the K2+G nucleotide substitution model. These analyses were conducted on the web server for ASAP (https://bioinfo.mnhn.fr/abi/public/asap/, accessed during November 2024). The resulting partition was determined by the lowest ASAP score. We also considered additional partitions when their ASAP score was identical to or close to the lowest value, as recommended (Puillandre et al. 2021). In such cases, we selected the closest partition to the delimitation predicted by phylogenetic analyses.

Hypothetical species delimitation was established by evaluating the phylogenetic analyses and the delimitation obtained through PTP and the ASAP partition. Therefore, the strains determined as unknown lineages were considered putative novel species and selected for further morphological studies.

### ﻿Morphological characterization

The strains determined as putative novel species through phylogenetic analyses were selected for further morphological analyses. Additional strains from curated collections were requested based on their morphological resemblance and close phylogenetic relationship to the marine strains (Table 1). We examined six strains of *Malbranchea* spp. available in the CBS (Westerdijk Fungal Biodiversity Institute, Utrecht, The Netherlands) and BCCM/IHEM (Sciensano, Brussels, Belgium) collections, labeled as *M. irregularis* (CBS 149937), *M. longispora* (CBS 135817), *M. multiseptata* (CBS 146931), *M. sinuata* (CBS 149938), *M. thaxteri* (CBS 319.61), and *Malbranchea* sp. (IHEM 28255). The former four species have been recently published from freshwater sediments and other non-aquatic substrates (Rodríguez-Andrade et al. 2021; Torres-Garcia et al. 2023).

Macroscopic characterization of the colonies was conducted using various culture media, i.e., PDA, OA, and phytone yeast extract agar (PYE; Pronadisa, Madrid, Spain). Prepubertal human hair was deposited on the surface of OA plates at 25 °C to induce sexual morph production for those strains related to *M. filamentosa* and susceptible to heterothallism (Sigler et al. 2002). Color notations in descriptions followed the *Methuen Handbook of Colour* (Kornerup and Wanscher 1967). Microscopic characterization was made on OA after 14 days at 25 °C in the dark, unless otherwise specified in the description. Reproductive structures were mounted with lactic acid and observed under an Olympus BH-2 bright-field microscope (Olympus Corporation, Tokyo, Japan). Descriptions were based on a minimum of 30 measurements of the relevant structures to provide size ranges. Photomicrographs were taken using a Zeiss Axio-Imager M1 light microscope (Oberkochen, Germany) with Nomarski (interference contrast) and phase contrast condensers, coupled with a DeltaPix Infinity digital camera. Photoplates were assembled using Photoshop CS6 v.13.0. Scanning electron microscopy (SEM) micrographs were obtained using a Quanta 600 FEG Scanning Electron Microscope (Thermo Fisher Scientific, Waltham, USA), and specimens were processed according to the protocol established by Figueras and Guarro (1988).

The strains isolated in this study were preserved in the culture collection of the Faculty of Medicine in Reus (FMR, Reus, Catalonia, Spain), and those representing novel fungi were deposited at the Westerdijk Fungal Biodiversity Institute (CBS collection), including holotypes as dry colonies on the most appropriate media for their sporulation. Nomenclatural novelties were registered in MycoBank (https://www.mycobank.org/, accessed in November 2024).

### ﻿Phylogeny and geographical distribution of related environmental sequences

Biogeographical distribution of the marine strains was assessed using the GlobalFungi database v.10.0, release version 5.0, accessed in December 2024 (Větrovský et al. 2020). This repository comprised data from 84,972 environmental samples originating from 846 studies (33% from Europe, 33% from Asia, 20% from North America, 4% from Australia, 2% from Africa, and 8% from other regions), encompassing terrestrial, airborne, aquatic, and marine samples. The database was organized with separate entries for the ITS1 and ITS2 regions, containing over 1 billion ITS1 sequences and more than 3 billion ITS2 sequences. First, we calculated the interspecific genetic distance of the ITS1 and ITS2 regions for each genus detected in marine sediments, following the methodology established for other fungal groups (Réblová et al. 2021b; Torres-Garcia et al. 2023). The resulting threshold, along with the full-coverage limit, was used to perform BLAST searches for each strain in GlobalFungi. Due to the large number of environmental sequences obtained, we selected representative sequences from a variety of geographical locations, biomes, and substrates. Sequence identifications were validated through phylogenetic analyses of the ITS1 and ITS2 regions separately. Metadata associated with each sample, including location, substrate, biome, mean annual temperature (MAT), mean annual precipitation (MAP), and pH, were used for ecological characterization. Geographic occurrences were represented using QGIS v.3.34.5 (QGIS Development Team 2024).

### ﻿Physiological tests

The ecological behavior of the onygenalean strains recovered from marine sediments was further tested *in vitro*, including the aforementioned strains from other collections. The physiological traits tested included salt tolerance and the ability to degrade cellulose, chitin, and keratin. Salt tolerance was assessed on MEA supplemented with 3.5, 10, 15, and 20% NaCl, using MEA as a negative control. The ability to degrade cellulose was evaluated on Cellulose Congo-Red Agar (CCA; 0.5 g KH_2_PO_4_, 0.25 g MgSO_4_, 2 g cellulose, 0.2 g Congo-Red, 2 g gelatin, 15 g agar, 1 L distilled water, pH 6.8–7.2) (Gupta et al. 2012). Chitinase activity was assessed using Chitin Agar (CA; 20 g colloidal chitin, 1 g (NH_4_)_2_SO_4_, 1 g K_2_HPO_4_, 0.5 g MgSO_4_, 0.5 g KCl, 0.5 g NaCl, 0.2 g CaCl_2_, 2 g FeSO_4_.7H_2_O, 15 g agar, 1 L distilled water) adjusted to pH 7 (Yilma and Kebede 2018). Colloidal chitin was obtained from commercial chitin (Sigma-Aldrich, Burlington, USA) through the modification of a published protocol (Hsu and Lockwood 1975). Briefly, 20 g of chitin was dissolved in 37% HCl by stirring at 40 °C, precipitated as a colloidal suspension by slowly adding 2 L of distilled water at 10 °C, collected by filtering through coarse filter paper, and washed with distilled water until pH 7. For these tests, colony diameter was measured after 21 days of incubation at 25 °C, and discoloration of Congo Red or CA was used as an indicator of positive cellulose- or chitin-degrading activity. Keratinophilic activity was tested by inoculating a suspension of fungal elements (ascospores, conidia, and hyphae) on a Petri dish containing 25 mL of distilled water, 1 mL of 10% yeast extract, and 1–1.5 cm of prepubertal child hair that had been previously washed with distilled water and sterilized (De Hoog et al. 2020). Plates were checked after 14 and 21 days, and the formation of perforating organs confirmed positive keratin degradation.

Finally, the ability of each putative novel species to grow at different temperatures was assessed on PDA from 5 to 45 °C at 5 °C intervals, including measurements at 37 °C, after 14 days of incubation. All physiological tests were performed in duplicate.

## ﻿Results

### ﻿Onygenalean strains isolated

We recovered 32 strains from the primary cultures of sediment samples that were identified morphologically as members of the order *Onygenales*. Most of these strains were recovered through the direct plating of the sediment, with only twelve strains (37.5%) detected through the SMF pre-treatment. Regarding the culture media of isolation, 16 strains were isolated from PDA+C, nine from SWMEA3%, six from DRBC, and one from SWOA. Combining morphology and comparison of the ITS barcode region, one strain was recognized as a member of *Myriodontium* in *Neoarthropsidaceae*, four strains as members of the genera *Aphanoascus* and *Byssoonygena* in *Onygenaceae*, eight strains as members of the genus *Malbranchea* in *Malbrancheaceae*, and 17 strains as members of the genera *Gymnascella*, *Gymnoascoideus*, *Gymnoascus*, *Narasimhella*, and *Sporendonema* in *Gymnoascaceae*. Nevertheless, six strains of *Malbranchea* (FMR 19403, FMR 19564, FMR 20150, FMR 20151, FMR 20852, and FMR 21121) and one strain of *Gymnoascoideus* (FMR 19992) did not match any described species in their respective genera. The only marine strain that could not be identified at the genus/family level was FMR 20744, which exhibited a sexual morph resembling the genus *Albidomyces*, whereas the asexual morph showed similarities to the genera *Amaurascopsis* and *Pseudoamaurascopsis* (Guarro et al. 1992a; Torres-Garcia et al. 2023). All these strains represented 22 species under the consolidated species concept, combining phylogeny, morphology, and ecology as explained in the following sections.

### ﻿General phylogenies

**Figure 2.**
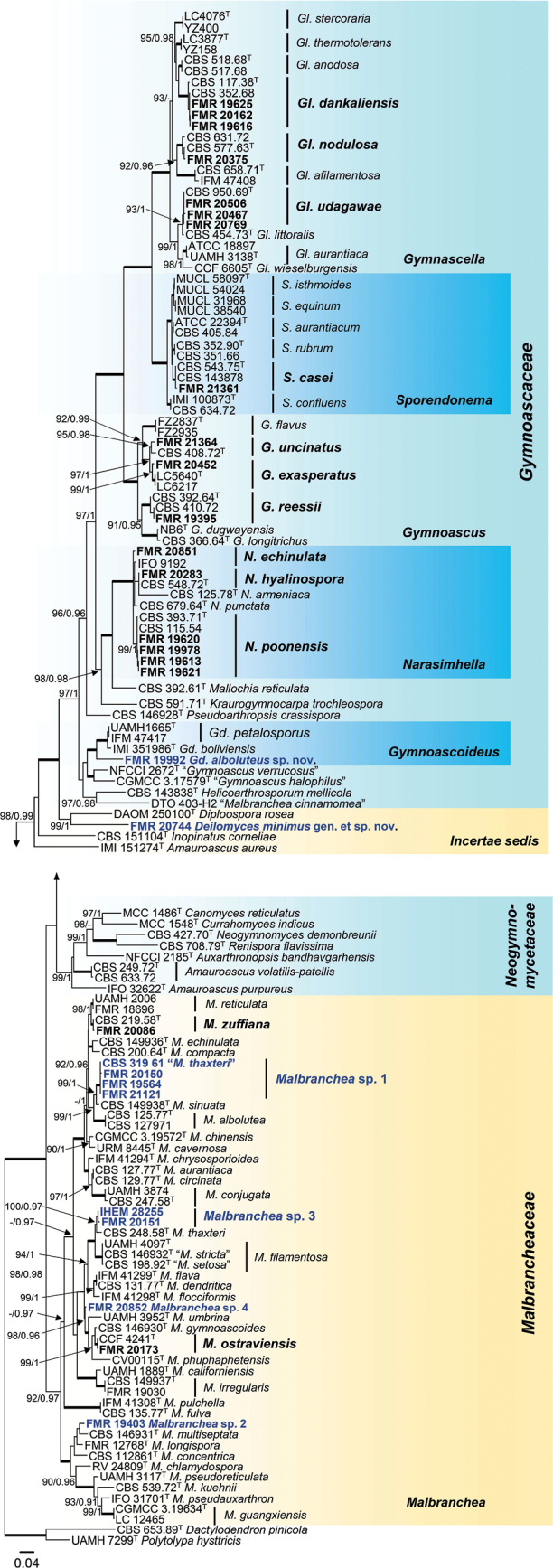
Phylogenetic tree inferred from a concatenated ITS–LSU alignment of 137 strains representing the families *Gymnoascaceae*, *Malbrancheaceae*, *Neogymnomycetaceae*, and a related *incertae sedis* clade in *Onygenales*. The tree is rooted to *Polytolypa hystricis* UAMH 7299 and *Dactylodendron pinicola* CBS 653.89. Numbers at the branches indicate support values (IQ-TREE-UFBS/BI-PP) above 90%/0.95. Fully supported branches (100/1) are highlighted in bold. Strains recovered in this work and the associated species are highlighted in bold. Quotation marks indicate strains with unresolved taxonomy. Putative novel species are highlighted in blue. Ex-type strains are indicated with T. The scale bar represents the expected number of changes per site.

The molecular identification and taxonomic position of the strains were assessed through phylogenetic analysis of the ITS and LSU regions. Each region was initially analyzed independently (Suppl. material 2: figs S1, S2), and after confirming the congruence between the topology of the resulting trees, both regions were concatenated into a single matrix. For the members of *Gymnoascaceae* and *Malbrancheaceae*, the final alignment encompassed 137 sequences representing ex-type and reference strains and also included members of *Neogymnomycetaceae* and a well-supported clade labeled as *incertae sedis* according to Kandemir et al. (2022). The outgroups selected were *Polytolypa hystricis* UAMH 7299 and *Dactylodendron pinicola* CBS 653.89. The alignment comprised 1,293 bp (700 bp for ITS and 593 bp for LSU), of which 584 positions were conserved (211 bp for ITS and 373 bp for LSU), and 671 were variable (465 bp for ITS and 206 bp for LSU). Among the variable sites, 544 were parsimony-informative (376 bp for ITS and 168 for LSU). The remaining positions included ambiguous or gap-only sites that were excluded from these categories. The models selected for the ML analysis were GTR+F+I+G4 for ITS and TIM3+F+R3 for LSU, while for the BI analysis, the best models were GTR+I+G for ITS and TIM1+I+G for LSU. The resulting phylogenetic tree confirmed the monophyly of the families included in the analyses with well-supported clades. Here, we represent the maximum-likelihood tree with the bootstrap support values of the ML analysis and the BI posterior probabilities at the nodes (Fig. 2). An additional phylogenetic tree was made to represent members of *Onygenaceae* and *Neoarthropsidaceae*, based on their great phylogenetic distance from the rest of the onygenalean strains recovered in this work (Suppl. material 2: fig. S3).

The strains recovered from the marine sediments were predominantly distributed in *Gymnoascaceae*, *Malbrancheaceae*, and *Onygenaceae*. The identification of each strain, along with its metadata, can be accessed in Table 1. In *Onygenaceae*, the following species were detected: Aphanoascus (A.) crassitunicatus (FMR 20176), *A. fulvescens* (FMR 20116, FMR 20522), and Byssoonygena (B.) ceratinophila (FMR 19558); while a single species was detected in *Neoarthropsidaceae*, Myriodontium (My.) keratinophilum (FMR 20839) (Suppl. material 2: fig. S3).

The family *Gymnoascaceae* was the most represented (Fig. 2), with the following species identified: Gymnascella (Gl.) dankaliensis (FMR 19616, FMR 19625, and FMR 20162), *Gl. nodulosa* (FMR 20375), *Gl. udagawae* (FMR 20467, FMR 20506, and FMR 20769); Gymnoascus (G.) reessii (FMR 19395); Narasimhella (N.) echinulata (FMR 20851), *N. hyalinospora* (FMR 20283), *N. poonensis* (FMR 19613, FMR 19620, FMR 19621, and FMR 19978); and Sporendonema (S.) casei (FMR 21361). However, the analysis of the ITS and LSU regions was inconclusive for the precise identification of the *Gymnoascus* strains FMR 20452 and FMR 21364. Therefore, we performed an additional concatenated ITS–LSU–*tub2* analysis of the genus, which confirmed these strains as *G. exasperatus* and *G. uncinatus*, respectively (Suppl. material 2: fig. S4).

In *Gymnoascaceae*, this ITS–LSU analysis resolved the strain FMR 19992 as an independent lineage in Gymnoascoideus (Gd.), phylogenetically related to the type strain of *Gd. boliviensis* (IMI 351986; 91% identity with ITS, 97% identity with LSU) and *Gd. petalosporus* (UAMH 1665; 91% identity with ITS, 97% identity with LSU). At the same time, the unidentified strain FMR 20744, together with the epitype strain of Diploospora (Di.) rosea (DAOM 250100), formed a well-supported *incertae sedis* clade in *Onygenales*. However, the former strain delineated an independent lineage with sufficient phylogenetic distance from *Di. rosea* (76% identity with ITS and 92% identity with LSU) to be considered a distinct taxon. Therefore, strain FMR 19992 is proposed as *Gd. alboluteus* sp. nov., and FMR 20744 is proposed as the novel genus Deilomyces (D.), typified with *D. minimus* gen. et sp. nov. The detailed morphological characterizations of these fungi are provided in the taxonomy section.

Among the Malbranchea (M.) marine strains, only FMR 20086 and FMR 20173 could be confirmed as *M. zuffiana* and *M. ostraviensis*, respectively. Since the ITS/LSU analysis was unable to confidently assign the remaining six strains to any known species, we carried out additional phylogenetic analyses for species delineation. The unidentified strains were labeled as *Malbranchea* sp. 1–4 in Fig. 2.

### ﻿*Malbranchea* phylogeny

**Figure 3.**
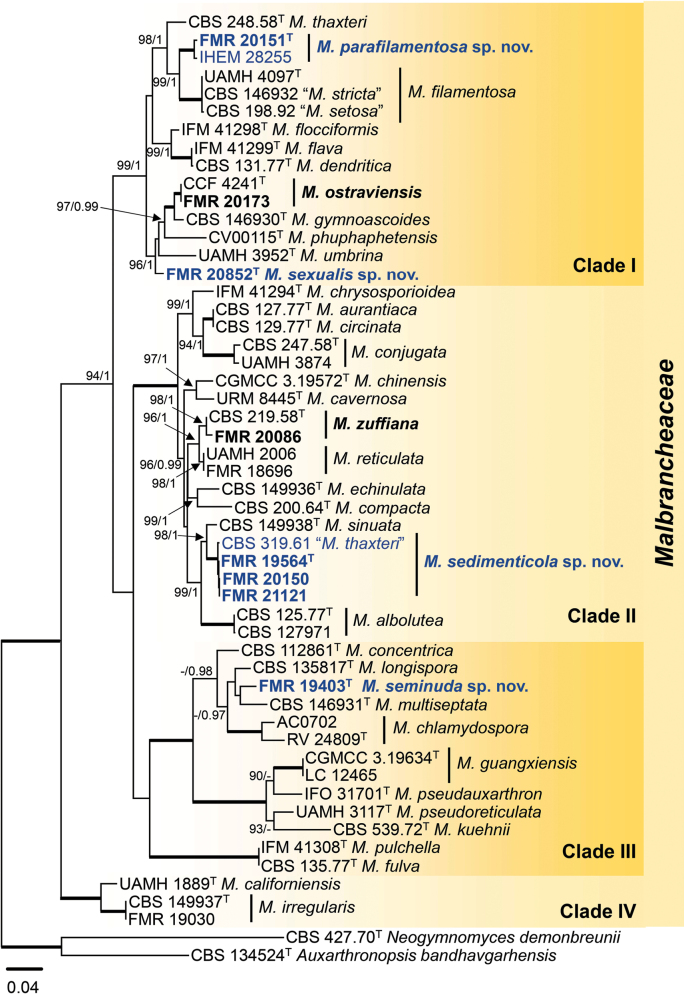
Phylogenetic tree inferred from a concatenated ITS–LSU–*tub2* alignment of 53 strains representing the genus *Malbranchea*. The tree is rooted to *Auxarthronopsis bandhavgarhensis* CBS 134524 and *Neogymnomyces demonbreunii* CBS 427.70. Numbers at the branches indicate support values (IQ-Tree-UFBS/BI-PP) above 90%/0.95. Fully supported branches (100/1) are highlighted in bold. Strains recovered in this work and the associated species are highlighted in bold. Quotation marks indicate strains with unresolved taxonomy. Putative novel species are highlighted in blue. Ex-type strains are indicated with ^T^. The scale bar represents the expected number of changes per site.

The concatenated ITS–LSU–*tub2* analysis resolved the phylogeny of the genus *Malbranchea* and delineated the relationships of the unidentified strains (Fig. 3). After confirming the congruence of the three regions, the final alignment comprised 1597 bp (631 bp for ITS, 581 bp for LSU, and 385 bp for *tub2*), of which 929 positions were conserved (312 bp for ITS, 447 bp for LSU, and 170 bp for *tub2*), and 635 were variable (302 bp for ITS, 127 bp for LSU, and 206 bp for *tub2*). Among the variable sites, 468 were parsimony-informative (216 bp for ITS, 101 bp for LSU, and 151 bp for *tub2*). The remaining positions included ambiguous or gap-only sites that were excluded from these categories. The models selected for the ML analysis were SYM+G4 for ITS, TNe+R2 for LSU, and TNe+I+G4 for *tub2*, while for the BI analysis, the best models were SYM+G for ITS, TIM1ef+I+G for LSU, and HKY+I+G for *tub2*. The outgroups selected were *Auxarthronopsis bandhavgarhensis* CBS 134524 and *Neogymnomyces demonbreunii* CBS 427.70. The representative strain of *M. cinnamomea*, DTO 403-H2, was excluded from the analysis based on its great phylogenetic distance from other *Malbranchea* species.

The resulting phylogenetic tree determined four major, highly supported clades in *Malbranchea*, where our strains were distributed across clades I, II, and III (Fig. 3). Each strain was compared with its closest phylogenetic taxa for the percentage of identity of the ITS, LSU, *tub2*, and *rpb2* regions, when available. In clade I, strain FMR 20151 was grouped with the unidentified *Malbranchea* strain (IHEM 28255), isolated from bat fur. These two strains represented an independent lineage phylogenetically related to the type strain of *M. thaxteri* (CBS 248.58; 97% identity with ITS, 93% identity with *tub2*, and 94% with *rpb2*) and the type strains of *M. filamentosa* (UAMH 4097; 97% identity with ITS) and *M. stricta* (CBS 146932; 97% identity with ITS, 99% identity with LSU). In the same clade, strain FMR 20852 formed a singleton lineage related to the type strains of *M. gymnoascoides* (CBS 146930; 97% identity with ITS, 99% identity with LSU), *M. ostraviensis* (CCF 4241; 97% identity with ITS, 96% identity with *tub2*), *M. phuphaphetensis* (CV00115; 92% identity with ITS, 99% identity with LSU, 96% identity with *tub2*), and *M. umbrina* (UAMH 3952; 94% identity with ITS, 99% identity with LSU, 96% identity with *tub2*). In clade II, the strains FMR 19564, FMR 20150, and FMR 21121 were grouped with strain CBS 319.61, which was labeled as *M. thaxteri*. These strains formed a well-supported clade related to the type strains of *M. sinuata* (CBS 149938; 98% identity with ITS, 99% identity with LSU, 96% identity with *tub2*, and 95% with *rpb2*) and *M. albolutea* (CBS 125.77; 96% identity with ITS, 99% identity with LSU, 93% identity with *tub2*), but were placed distantly from the type strain of *M. thaxteri* included in clade I. In clade III, strain FMR 19403 formed a singleton, distant lineage related to the type strains of *M. concentrica* (CBS 112861; 92% identity with ITS, 94% identity with *tub2*), *M. longispora* (CBS 135817; 95% identity with ITS, 99% identity with LSU, 96% identity with *tub2*), and *M. multiseptata* (CBS 146931; 94% identity with ITS, 99% identity with LSU). These marine lineages appeared to represent four putative novel species based on their substantial phylogenetic distances from their respective closest species. This hypothesis was further tested through speciation analyses.

The ASAP and bPTP speciation analyses were performed on both the concatenated and single ITS alignments of *Malbranchea* (Suppl. material 2: fig. S5). Both the concatenated and ITS ASAP tests determined each marine lineage as an independent partition, and the results for the other *Malbranchea* species were congruent with the phylogenetic analysis. For the bPTP tests, the resulting topology for the concatenated alignment also recognized each marine lineage as independent. However, the posterior probabilities for speciation were low in clades expected to represent speciation events, suggesting that our data might not conform well to the software’s algorithm.

Therefore, based on phylogenetic evidence, morphological features, and the ecological data presented below, we concluded that *Malbranchea* sp. 1–4 represented novel species within the genus. These are proposed as *M. parafilamentosa* sp. nov. (FMR 20151 and IHEM 28255; *Malbranchea* sp. 3 in Fig. 2), *M. sedimenticola* sp. nov. (FMR 19564, FMR 20150, FMR 21121, and CBS 319.61; *Malbranchea* sp. 1 in Fig. 2), *M. seminuda* sp. nov. (FMR 19403; *Malbranchea* sp. 2 in Fig. 2), and *M. sexualis* sp. nov. (FMR 20852; *Malbranchea* sp. 4 in Fig. 2). A detailed morphological characterization of these fungi is provided in the taxonomy section.

### ﻿Species distribution across the sampled areas

The majority of the onygenalean species isolated in this study were recovered from Arrabassada and Miracle beaches and from the discharge area of the Ebro River (Table 1).

At Arrabassada Beach, nine species were identified, with *A. crassitunicatus*, *Gd. alboluteus*, *M. ostraviensis*, *M. parafilamentosa*, and *N. hyalinospora* exclusively detected at this location. Four species were recovered only from Miracle Beach, i.e., *B. ceratinophila*, *G. reessii*, *M. seminuda*, and *M. zuffiana*. Three additional species—*Gl. dankaliensis*, *N. poonensis*, and the novel species *M. sedimenticola*—were detected at both Arrabassada and Miracle beaches. *Aphanoascus fulvescens* was found at Arrabassada Beach and in the discharge area of the Ebro River. The sediments collected in the Ebro River area revealed a distinct community of onygenalean fungi, primarily dominated by members of *Gymnoascaceae*. This group was represented by *G. exasperatus*, *N. echinulata*, and two species of *Gymnascella*—*Gl. nodulosa* and *Gl. udagawae*—along with the novel species *M. sexualis*. In the discharge area of the Llobregat River, we detected *G. uncinatus*, *M. sedimenticola*, and *S. casei*. In contrast, only two species were identified in the Ter River discharge area: *My. keratinophilum* and the novel taxon *D. minimus*.

These species were predominantly (77%) recovered from sediments collected at depths between 27 and 30 m. Notably, *N. poonensis*, *Gl. dankaliensis*, and *Gl. udagawae*—the most prevalent species in our study (n = 4, n = 3, and n = 3, respectively)—were almost exclusively detected at 27 m depth. Although the novel species were less abundant, they were also primarily recovered from the deepest sediments. The only exceptions were *M. sedimenticola*, also recovered from sediments at 19 m depth and from soil (CBS 319.61), and *M. parafilamentosa*, also isolated from bat fur (IHEM 28255). Based on this substrate variability, we assessed geographic and substrate distribution using the GlobalFungi database.

### ﻿Global biogeography of the species identified

Prior to biogeographic analysis, environmental sequences were clustered with curated sequences in individual alignments. Environmental sequences were obtained from GlobalFungi using a threshold we determined at 98% interspecific distance for the ITS1 and ITS2 regions of *Aphanoascus*, *Byssoonygena*, *Gymnoascoideus*, *Gymnoascus*, *Gymnascella*, *Malbranchea*, *Myriodontium*, *Narasimhella*, and *Sporendonema*. For the novel genus *Deilomyces*, we used a 95% identity threshold, based on its great distance to its sister lineage (*Diploospora*) and the poor representation of these taxa in public databases. BLAST searches on GlobalFungi were conducted according to these thresholds, and the selected environmental sequences were included in the alignments to ensure phylogenetic identification (Suppl. material 2: figs S6–S17). The species with the highest number of environmental sequences were *A. fulvescens* (Suppl. material 2: fig. S6), *Gl. dankaliensis* (Suppl. material 2: fig. S9), *G. reessii* (Suppl. material 2: figs S11, S12), and *My. keratinophilum* (Suppl. material 2: fig. S15). Additionally, the phylogenetic analysis of the ITS2 region of *Gymnoascoideus* revealed three environmental sequences that clustered as an independent hidden taxon, labeled ITS2-ENV1 (Suppl. material 2: fig. S10). Unfortunately, these sequences were not detected in the ITS1 region, where only a single sequence was related to strain FMR 19992 (Suppl. material 2: fig. S10). For the genus *Narasimhella*, only the ITS1 region was used in the biogeography analysis (Suppl. material 2: fig. S18). This region detected environmental sequences exclusively associated with *N. poonensis*. Other species in the genus—*N. armeniaca*, *N. echinulata*, *N. hyalinospora*, and *N. punctata*—showed close phylogenetic relationships, making confident assignment of environmental sequences impossible. Analysis of the ITS2 region revealed a similar pattern (identity ≥ 98% between species), so ITS2 was excluded from the biogeographic analysis.

**Figure 4.**
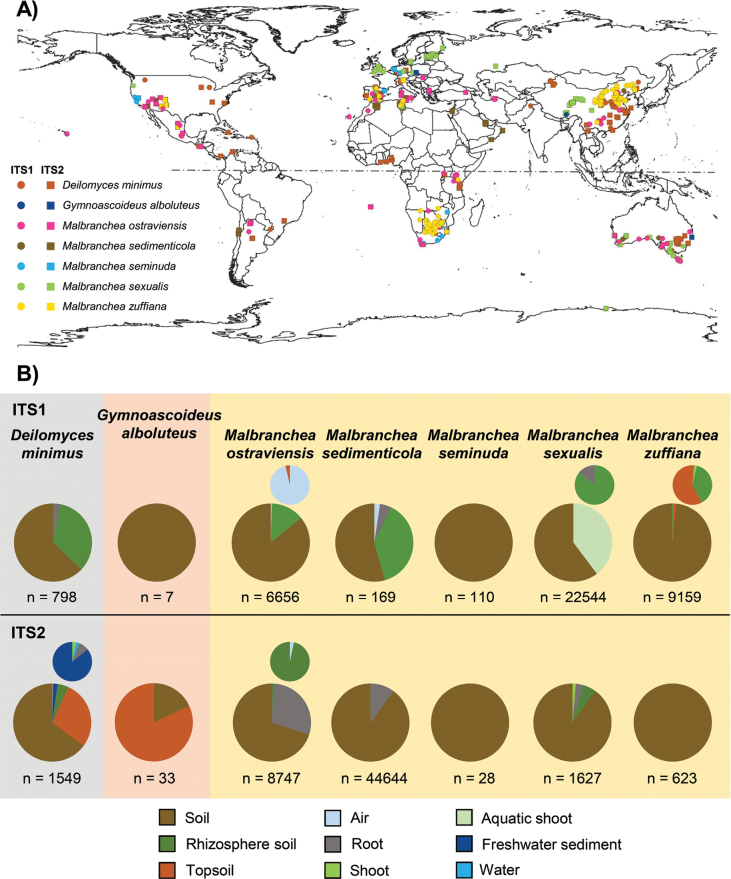
Biogeographical data extracted from the GlobalFungi v.10.0 (release version 5.0) database (accessed in December 2024). A. Geographic distribution of ITS1 and ITS2 environmental sequences representing the species of *Deilomyces*, *Gymnoascoideus*, and *Malbranchea* recovered from marine sediments in this study. B. Pie charts representing the substrates where each species has been detected. When rare substrates are masked in the pie chart, an additional chart is provided excluding the major substrates.

The search on GlobalFungi revealed that some species appeared to represent common fungi with a worldwide distribution, while others were rare species with a more restricted biogeography. Most species of *Malbranchea*, as well as *A. fulvescens*, *D. minimus*, *G. reessii*, *Gd. alboluteus*, and *My. keratinophilum*, were detected in both hemispheres (Fig. 4A; Suppl. material 2: fig. S18). *Deilomyces minimus* was found to be a frequent fungus (more than 1,500 observations), particularly in tropical areas of America, Africa, and Asia (Fig. 4A). For *Malbranchea*, *M. ostraviensis* and *M. zuffiana* were the most common species across different environmental samples, with *M. zuffiana* clustering in South Africa and eastern China (Fig. 4A). The novel species *M. sexualis* and *M. sedimenticola* showed a narrower range of distribution, and *M. seminuda* was detected as a rare fungus (fewer than 150 observations), with a geographic distribution restricted to the area between 30°–40° North and South (Fig. 4A). A similar pattern of restricted geographical distribution was also observed in *Gymnoascoideus*. The novel species *Gd. alboluteus* was also a rare fungus (fewer than 50 observations), detected only in Europe and Australia (Fig. 4A). The novel species *M. parafilamentosa*, recovered from marine sediments and bat fur (Table 1), represented another rare fungus that, in this case, could not be associated with any environmental sequence in GlobalFungi.

The species detected in GlobalFungi were predominantly terrestrial, primarily associated with soil-related substrates such as bulk soil, rhizosphere soil, topsoil, and plant roots (Fig. 4B). The species that exhibited wide geographic distribution appeared to endure a variety of environmental conditions. In particular, *D. minimus*, *M. ostraviensis*, and *M. zuffiana* were predominantly detected in soil-related samples from croplands and woodlands of temperate to tropical regions (Fig. 4A, B). These areas were characterized by mean annual temperatures (MATs) between 4–20 °C and mean annual precipitations (MAPs) between 200–1,000 mm, including North America, the Caribbean, southern Europe, central and southern Africa, eastern China, and Australia. *Malbranchea sexualis* was also predominantly associated with soil samples from temperate and tropical areas in Europe, North America, China, and Australia (Fig. 4A, B), but it endured colder MATs between -5 °C and 20 °C. Among the species with more restricted geographic distribution, *M. seminuda* was exclusively detected in soil samples from grasslands with MATs around 15 °C and MAPs around 700 mm in southern Europe, the western United States, and southern Africa (Fig. 4A, B). *Malbranchea sedimenticola* was associated with soil-related substrates from deserts and shrublands with MATs between 7 °C and 20 °C and MAPs between 20 mm and 500 mm, on the west coast of the American continent, southern Europe, western Australia, and northern Africa (Fig. 4A, B). For the species of *Aphanoascus*, *Byssoonygena*, *Gymnascella*, *Gymnoascus*, *Myriodontium*, and *Sporendonema*, the geographical distribution observed in GlobalFungi predominantly covered terrestrial areas and was also associated with soil-related substrates. Among them, *A. fulvescens* and *G. reessii* were the most abundant and prevalent species across terrestrial samples (Suppl. material 2: figs S18, S19).

Nevertheless, several species were also detected in water-related substrates (Fig. 4B; Suppl. material 2: fig. S19). The species *Gl. nodulosa*, *M. sexualis*, and *S. casei* were strongly associated with aquatic plant shoots. Freshwater sediment was another common water-related substrate, with *A. crassitunicatus*, *Gl. dankaliensis*, *Gl. udagawae*, *G. reessii*, and *N. poonensis* detected in China (Suppl. material 2: figs S18, S19), and *D. minimus* in Australia (Figs 4A, B). Finally, marine sediments represented an unusual substrate, but the species *A. fulvescens*, *Gl. dankaliensis*, and *My. keratinophilum* were detected in the Pacific Ocean, and *G. reessii* in the Atlantic Ocean (Suppl. material 2: figs S18, S19).

### ﻿Physiology

**Figure 5.**
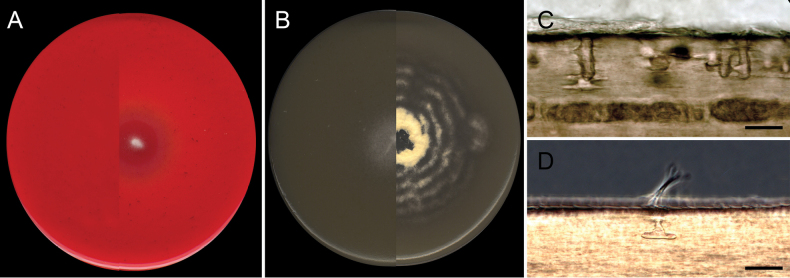
Interpretation of cellulolytic, chitinolytic, and keratinolytic activities after 21 days. A. Cellulose Congo-Red Agar: no inoculation on the left side, positive degradation on the right side (*Malbranchea sedimenticola*FMR 19564). B. Chitin Agar: negative on the left side (*Byssoonygena ceratinophila*FMR 19558), positive on the right side (*Myriodontium keratinophilum*FMR 20839). C. Perforating organs in prepuberal human hair (*B. ceratinophila*FMR 19558). D. Hyphae penetrating human hair and perforating organ extension under phase contrast microscopy (*B. ceratinophila*FMR 19558). Scale bars: 25 µm.

Based on the dominant terrestrial prevalence detected through GlobalFungi for the species recovered from marine sediments, we aimed to test *in vitro* their ability to grow under different conditions resembling both terrestrial and marine environments. The results of the physiological tests are represented in Table 2 and Fig. 5. Most strains grew in media supplemented with up to 10% NaCl. Only the strains FMR 19625 (*Gl. dankaliensis*), FMR 19564 (*M. sedimenticola*), CBS 149938 (*M. sinuata*), and FMR 20151 (*M. parafilamentosa*) tolerated up to 15% NaCl, and *My. keratinophilum* (FMR 20839) was the only species that grew up to 20% NaCl. In contrast, the strains of *D. minimus* (FMR 20744), *M. seminuda* (FMR 19403), and *M. multiseptata* (CBS 146931) did not grow in any media supplemented with NaCl. Regarding the test on cellulose degradation, all strains grew on CCA and were able to degrade cellulose, producing a halo around the colony. For the test assessing chitin degradation, all strains managed to grow on CA, except *D. minimus*. However, degradation halos were only observed in the strains of *M. parafilamentosa* (FMR 20151 and IHEM 28255), *M. sexualis* (FMR 20852), *M. sinuata* (FMR 18266), *My. keratinophilum* (FMR 20839), and *S. casei* (FMR 21361). Finally, all strains grew in the keratin test, but hair degradation was only observed in the strains of *A. crassitunicatus* (FMR 20176), *A. fulvescens* (FMR 20116, FMR 20522), and *B. ceratinophila* (FMR 19558). The production of perforating organs can be observed in Fig. 5C, D.

**Table 2.** Performance of the strains studied in this work on different physiological tests after 21 days of incubation.

Taxon name	Strain	Colony growth (mm)					Halo zone^1^				
							Cellulose		Chitin		Hair degradation
		MEA	MEA 3.5%	MEA 10%	MEA 15%	MEA 20%	Growth	Halo	Growth	Halo	Perforating organs
*Aphanoascus crassitunicatus*	FMR 20176	85	10	1	0	0	44	+	40	–	+
*Aphanoascus fulvescens*	FMR 20116	85	26	0	0	0	42–46	+	20–28	–	+
	FMR 20522	85	20–24	1	0	0	45	+	45	–	+
*Byssoonygena ceratinophila*	FMR 19558	70	3	0	0	0	24	+	20–28	–	+
*Deilomyces minimus*	FMR 20744	8–9	0	0	0	0	1	+	0	–	–
*Gymnascella dankaliensis*	FMR 20162	46–52	85	0	0	0	63–65	+	84	–	–
	FMR 19625	50–52	84	50–52	7	0	36–40	+	84	–	–
	FMR 19616	48–50	84	32–34	0	0	40–47	+	84	–	–
*Gymnascella nodulosa*	FMR 20375	62–66	56	24–26	0	0	26–30	+	84	–	–
*Gymnascella udagawae*	FMR 20467	54–55	63–64	0	0	0	72–75	+	84	–	–
	FMR 20506	50–52	60	0	0	0	26–39	+	84	–	–
	FMR 20769	84	5	0	0	0	84	+	84	–	–
*Gymnoascoideus alboluteus*	FMR 19992	30	32	5	0	0	8–10	+	40	–	–
*Gymnoascus exasperatus*	FMR 20452	24–26	43–45	1–2	0	0	24	+	46–48	–	–
*Gymnoascus reessii*	FMR 19395	58–60	44–48	1	0	0	56–58	+	84	–	–
*Gymnoascus uncinatus*	FMR 21364	38	66	0	0	0	38–40	+	25	–	–
*Malbranchea irregularis*	FMR 19016	13–15	10	0	0	0	18–20	+	25–30	–	–
*Malbranchea longispora*	CBS 135817	63–66	1–2	0	0	0	48–51	+	76–80	–	–
*Malbranchea multiseptata*	CBS 146931	52–56	0	0	0	0	26–32	+	31	–	–
*Malbranchea ostraviensis*	FMR 20173	36–38	26	0	0	0	5–18	+	56–58	–	–
*Malbranchea sinuata*	FMR 18266	38–45	45–47	10–12	3	0	43–45	+	84	+	–
*Malbranchea sedimenticola*	FMR 19564	53–55	40–41	9–10	3	0	15–18	+	80–84	–	–
	FMR 20150	48–51	28–29	3–4	0	0	11	+	84	–	–
	FMR 21121	46–50	38–40	5	0	0	8	+	75–78	–	–
	CBS 319.61	55–60	35	5	0	0	10–15	+	84	–	–
*Malbranchea seminuda*	FMR 19403	33–36	0	0	0	0	25–32	+	58–64	–	–
*Malbranchea parafilamentosa*	FMR 20151	10–12	12–14	10–11	5	0	8	+	28–30	+	–
	IHEM 28255	10	20–21	9	0	0	8	+	30	+	–
*Malbranchea sexualis*	FMR 20852	12	17	8–10	0	0	20–22	+	24–26	+	–
*Malbranchea zuffiana*	FMR 20086	26–27	47–50	10	0	0	70–72	+	84	–	–
*Myriodontium keratinophilum*	FMR 20839	50	60	12	4	2	26–28	+	55	+	–
*Narasimhella echinulata*	FMR 20851	84	84	36–40	0	0	52	+	84	–	–
*Narasimhella hyalinospora*	FMR 20283	38–40	23–25	15	0	0	62	+	84	–	–
*Narasimhella poonensis*	FMR 19978	84	84	24–30	0	0	62–64	+	84	–	–
	FMR 19613	72–85	76–78	19	0	0	80	+	84	–	–
	FMR 19621	84	84	16–18	0	0	63–67	+	84	–	–
	FMR 19620	84	84	30–32	0	0	78–80	+	84	–	–
*Sporendonema casei*	FMR 21361	12–15	20	7–9	0	0	20	+	56–60	+	–

^1^(+) indicates positive degradation activity and the production of a degradation halo or perforating organs; (–) indicates the absence of degradation.

### ﻿Taxonomy

#### ﻿ Deilomyces

Taxon classificationAnimaliaOnygenalesGymnoascaceae

Guerra-Mateo, Gené & Cano gen. nov.

A5C9A91F-D80B-548F-A4C0-2A2E72748845

856502

##### Etymology.

Greek *deilós* (δειλός), timid, and Greek *múkēs* (μύκης), fungus, referring to the slow growth in culture and restricted production of ascomata.

##### Classification.

*Eurotiomycetes*, *Onygenales*, *Incertae sedis*

##### Type species.

*Deilomyces minimus* Guerra-Mateo, Gené & Cano

##### Description.

***Mycelium*** composed of hyaline, septate, branched, smooth-walled hyphae. ***Asexual morph***Chrysosporium-like. ***Conidia*** thallic, terminal, intercalary, occasionally lateral, hyaline to subhyaline, smooth-walled to tuberculate, thick-walled; ***terminal and lateral conidia*** aseptate, subglobose to obpyriform or short clavate with truncated base; ***intercalary conidia*** alternate, frequently disposed in a knuckle joint position, 0–1-septate, subglobose, subcylindrical, obpyriform, or short clavate with truncated base. ***Sexual morph*** with gymnothecial ascomata. ***Gymnothecia*** superficial, single or aggregated, globose or subglobose; peridium composed of a subtle network of septate, branched, anastomosed, hyaline, smooth- and thick-walled hyphae; peridial appendages absent. ***Asci*** 8-spored, evanescent, globose, subglobose, or pyriform. ***Ascospores*** unicellular, smooth- and thick-walled, globose, subglobose, ellipsoidal, or irregularly shaped.

#### ﻿ Deilomyces minimus

Taxon classificationAnimaliaOnygenalesGymnoascaceae

Guerra-Mateo, Gené & Cano sp. nov.

60309B22-B242-57AA-96C5-291380F88D23

856503

Fig. 6

##### Etymology.

Latin *minimus*, small, referring to the small size of reproductive structures.

**Figure 6.**
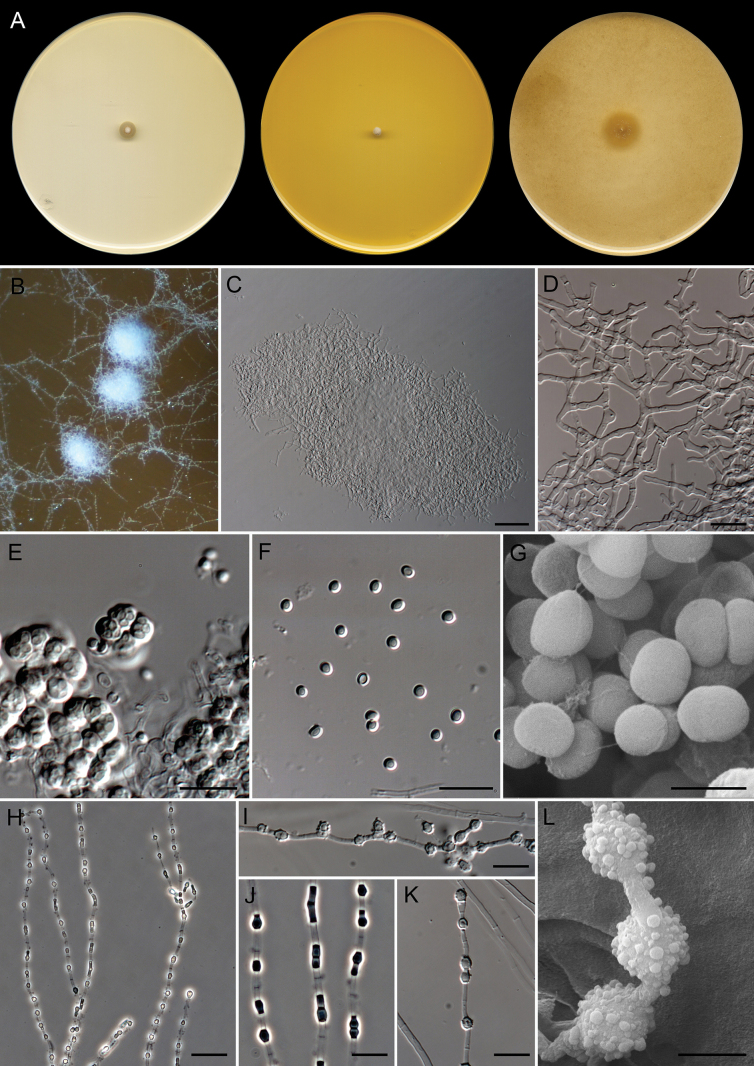
*Deilomyces minimus* (ex-type FMR 20744). A. Colonies on PDA, PYE, and OA after 14 days at 25 °C. B, C. Ascomata. D. Peridial hyphae. E. Asc. F. Ascospores under light microscopy. G. Ascospore surface under SEM. H–K. Fertile hyphae and arthroconidia. L. Arthroconidia under SEM. Scale bars: 50 µm (C); 10 µm (D–F, H–K); 2 µm (G); 2.5 µm (L).

##### Type.

Spain • Catalonia, Mediterranean coast, Estartit, discharging area of the Ter River, 42°1'34"N, 3°13'7"E, from sediments at 27 m depth, April 2023, D. Guerra-Mateo & J. Gené (holotype CBS H-25613; cultures ex-type FMR 20744, CBS 152721).

##### Description.

***Saprobic*** on marine sediments. ***Mycelium*** immersed and superficial, composed of hyaline, septate, branched, smooth-walled, 1.5–2 µm wide hyphae. ***Asexual morph***Chrysosporium-like. ***Conidia*** thallic, terminal or intercalary, occasionally lateral on short stalks, hyaline to subhyaline, smooth-walled to tuberculate, thick-walled; ***terminal and lateral conidia*** on straight or slightly curved branches, aseptate, subglobose, obpyriform, or short clavate with truncated base, 3–4.5 × 2.5–3 µm; ***intercalary conidia*** alternate, frequently disposed in a knuckle joint position, 0–(1)-septate, constricted at septum, subglobose, subcylindrical, obpyriform, or short clavate with truncated base, 5.5–8.5(–10.5) × 2.5–3 µm. ***Sexual morph*** with gymnothecial ascomata. ***Gymnothecia*** superficial, single or aggregated, initially white, turning pale yellow at maturity, globose or subglobose, 150–550 µm diam.; peridium composed of a subtle network of septate, branched, anastomosed, hyaline, smooth- and thick-walled, cylindrical, 1.5–2 µm wide hyphae; peridial appendages absent. ***Asci*** 8-spored, evanescent, globose, subglobose, or pyriform, 4.5–6 × 4–5 µm. ***Ascospores*** unicellular, pale yellow, smooth- and thick-walled, globose, subglobose, ellipsoidal, or irregularly shaped, 2.5 × 1.5–2.5 µm.

##### Culture characteristics

(after 14 days at 25 °C). Colonies on OA reaching 13–16 mm diam., flat, predominantly submerged, aerial mycelium sparse, with scattered white (1A1) ascomata towards periphery, margin diffuse; reverse uncolored; producing a conspicuous urea odor. On PDA, 5–7 mm diam., slightly umbonate, floccose and white (1A1) at center, velvety and yellowish white (4A2) towards periphery, margin diffuse; reverse uncolored. On PYE, 3–4 mm diam., umbonate, woolly, white (1A1), margin feathery; reverse uncolored. Diffusible pigment not observed in any of the media studied.

##### Cardinal temperatures for growth.

Minimum 20 °C (3 mm), optimum 25 °C (7 mm), and maximum 30 °C (3 mm).

##### Habitat and geographic distribution.

Marine sediments in Spain. In GlobalFungi, in soil from different environments (forests, woodland, grassland, wetland, cropland, and urban), water, rhizosphere soil, plant shoots, roots, and freshwater sediments. Argentina, Australia, Brazil, China, Colombia, Cuba, Germany, Italy, Nigeria, Panama, South Africa, Tanzania, and the USA (Fig. 5).

##### Notes.

*Deilomyces minimus* is phylogenetically related to *Diploospora rosea* (Fig. 2). These two fungi represent two genera based on the great phylogenetic distance between them and the distinct pattern of conidiogenesis; while *D. minimus* shows a Chrysosporium-like asexual morph with thallic conidia, *Di. rosea* displays a mixture of blastic and thallic conidia arising in acropetal, sometimes basipetal, chains directly from the hyphae (Tanney et al. 2015). The asexual morph of *D. minimus* resembles that of Amaurascopsis (As.) perforata (Guarro et al. 1992a), Polytolypa (P.) hystricis (Scott et al. 1993), and Pseudoamaurascopsis (Pd.) spiralis (Torres-Garcia et al. 2023). These three species represent an *incertae sedis* lineage in *Onygenales*, defined as clade X by Torres-Garcia et al. (2023). These species are characterized by the production of Chrysosporium-like thallic, terminal, and intercalary, smooth-walled or tuberculate conidia. *Deilomyces minimus* can be distinguished from *As. perforata* by smaller terminal conidia (3–4.5 × 2.5–3 µm vs. (3)4–6.5(7.5) × 2.5–4.5 µm) (Guarro et al. 1992a), from *Pd. spiralis* by shorter intercalary conidia [5.5–8.5(–10.5) µm long vs. 7–15(–22.5) µm long] (Torres-Garcia et al. 2023), and from *P. hystricis* by the conidial ornamentation (smooth to tuberculate vs. smooth) (Scott et al. 1993). In addition, the sexual morph of *D. minimus* resembles those of the phylogenetically distant species of the family *Neoarthropsidaceae*, Albidomyces (Al.) albicans and Neoarthropsis (Ne.) sexualis. These species produce gymnothecial ascomata composed of a conspicuous network of hyaline to pale yellow and smooth hyphae, subglobose to pyriform, evanescent asci, and globose or subglobose ascospores. *Deilomyces minimus* can be distinguished from *Ne. sexualis* by smaller ascomata and thinner peridial hyphae (150–550 µm vs. 365–810 µm diam., and 1.5–2 µm vs. 3–4 µm wide, respectively), and differs from *Al. albicans* by smaller asci and ascospores (4.5–6 × 4–5 µm vs. 8–11 × 11–17 µm, and 2.5 × 1.5–2.5 µm vs. 3–5 × 3–4.5 µm, respectively). Moreover, the ascospores of *D. minimus* are smooth, while those of *Ne. sexualis* and *Al. albicans* are punctate and punctate to reticulate, respectively (Torres-Garcia et al. 2023).

#### ﻿ Gymnoascoideus alboluteus

Taxon classificationAnimaliaOnygenalesGymnoascaceae

Guerra-Mateo, Cano & Gené sp. nov.

9AD9FE9B-521F-58D4-8C48-5522DAE3D345

859155

Fig. 7

##### Etymology.

Referring to the white colonies with yellowish shades produced in culture.

**Figure 7.**
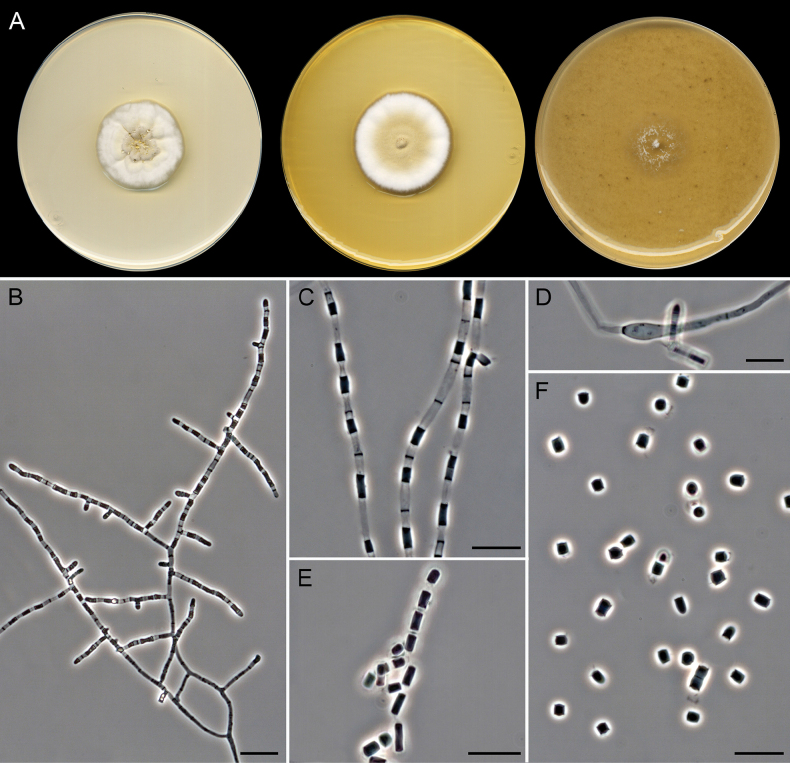
*Gymnoascoideus alboluteus* (extype FMR 19992). A. Colonies on PDA, PYE, and OA after 14 days at 25 °C. B, C. Fertile hyphae. D. Racket hyphae. E. Arthroconidial release. F. Arthroconidia. Scale bars: 20 µm (B); 10 µm (C, E, F); 5 µm (D).

##### Type.

Spain • Catalonia, Mediterranean coast, Tarragona, Platja de la Arrabassada, 41°6'45"N, 1°16'51"E, from sediments at 27 m depth, March 2022, G. Quiroga-Jofre & D. Guerra-Mateo (holotype CBS H-25762, ex-type FMR 19992, CBS 154002).

##### Description.

***Saprobic*** on marine sediments. ***Mycelium*** superficial and immersed, composed of hyaline, septate, branched, smooth-walled, 2.5–3 µm wide hyphae. ***Asexual morph*** with straight, branched, cylindrical, 2.5–3 µm wide fertile hyphae, giving rise to arthroconidia in a random disposition. ***Arthroconidia*** enteroarthric, 0–1-septate, hyaline, smooth- and thin-walled, cylindrical to subcylindrical, barrel- or T-shaped, campaniform, with conspicuous frills, (2.5–)3.5–6(–9) × 2.5–3 µm; secession rhexolytic. ***Racket hyphae*** present. ***Sexual morph*** not observed.

##### Culture characteristics

(after 14 days at 25 °C). Colonies on OA reaching 24–26 mm diam., flat with sparse aerial mycelium, white (1A1), margin entire and submerged; reverse uncolored. On PDA, 30 mm diam., crateriform, slightly elevated, radially sulcate, fasciculate in center to velvety towards periphery, white (1A1) with light yellow (3A4) exudate in center, margin entire; reverse uncolored. Diffusible pigment light yellow (3A4). On PYE, 36 mm diam., slightly elevated, radially sulcate, woolly or floccose, yellowish grey (4B3) to white (1A1), margin fimbriate; reverse radially sulcate, olive brown (4D8) to white (1A1).

##### Cardinal temperatures for growth.

Minimum 5 °C (2 mm), optimum 25 °C (30 mm), maximum 35 °C (1 mm).

##### Habitat and geographic distribution.

Marine sediments in Spain. In GlobalFungi, in soil from forests and wetlands. Australia, China, and the Czech Republic (Fig. 4).

##### Notes.

*Gymnoascoideus alboluteus* represents an independent lineage phylogenetically related to *Gd. petalosporus* and *Gd. boliviensis* (Fig. 3). Macroscopically, these species differ in colony coloration on OA and PYE; *Gd. petalosporus* displays white colonies with buff or greenish-brown shades (Orr et al. 1977), while in *Gd. alboluteus* they are white with yellowish areas, and *Gd. boliviensis* exhibits colonies in orange shades (Guarro et al. 1992b). Microscopically, the three species produce the asexual morph, which is characterized by fertile hyphae that give rise to arthroconidia randomly. The morphological differences between their asexual morphs are the following: *Gd. petalosporus* displays buff or brown arthroconidia of 2.1–11.9 × 1.4–5.6 µm (Orr et al. 1977), *Gd. alboluteus* produces hyaline arthroconidia of 2.5–9 × 2.5–3 µm, and in *Gd. boliviensis*, they are also hyaline but slightly smaller (2–7 × 2–2.5 µm) (Guarro et al. 1992b). Another difference is the lack of racket hyphae in the latter species, which are present in *Gd. petalosporus* and *Gd. alboluteus*. In addition, *Gd. petalosporus* and *Gd. boliviensis* produce sexual morphs in culture. Although we have not observed the sexual morph for *Gd. alboluteus* in any of the media tested, the great phylogenetic distance to other species of the genus confirms it as a novel species.

#### ﻿ Malbranchea parafilamentosa

Taxon classificationAnimaliaOnygenalesGymnoascaceae

Guerra-Mateo, Cano & Gené sp. nov.

C258B588-FEF6-5664-86FA-9D03CFBA416E

856498

Fig. 8

##### Etymology.

Greek *para*- (*παρα*-), to resemble, referring to the morphological similarity to the sexual and asexual morphs of *M. filamentosa*.

**Figure 8.**
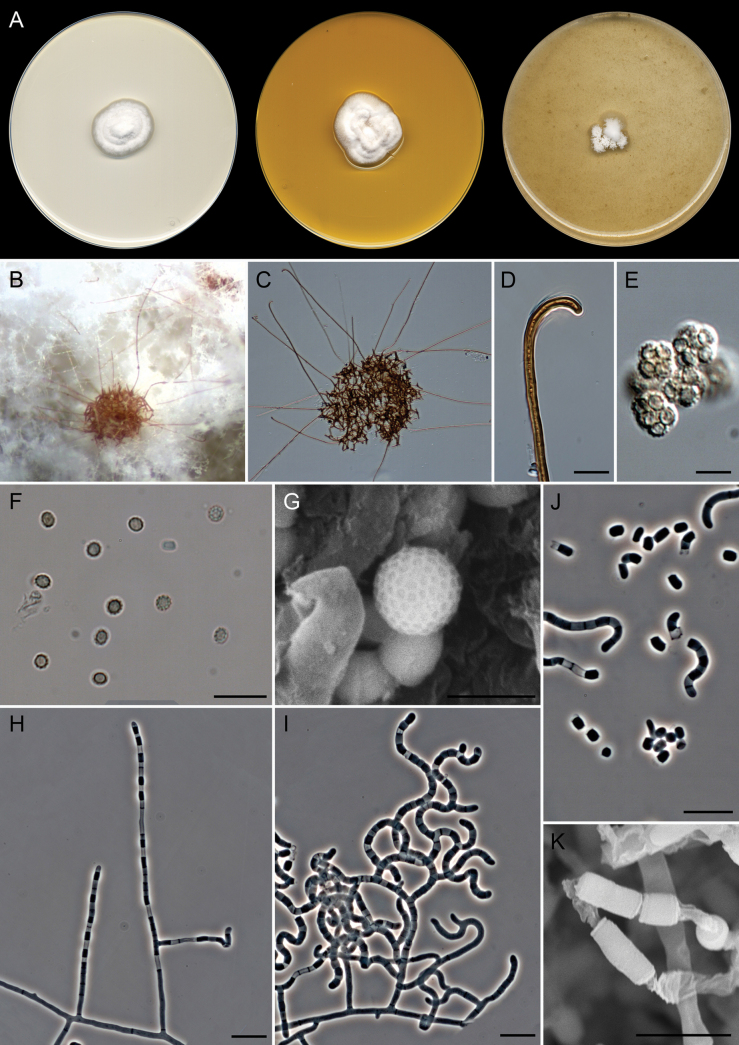
*Malbranchea parafilamentosa* (ex-type FMR 20151). A. Colonies on PDA, PYE, and OA after 14 days at 25 °C. B, C. Ascomata. D. Hooked appendage tip. E. Asci. F. Ascospores under light microscopy. G. Ascospore surface under SEM. H–J. Fertile hyphae and arthroconidia under light microscopy. K. Arthroconidia under SEM. Scale bars: 10 µm (D, F, H–J); 5 µm (E, G, K).

##### Type.

Spain • Catalonia, Mediterranean coast, Tarragona, Platja de la Arrabassada, 41°6'45"N, 1°16'51"E, from sediments at 27 m depth, March 2022, G. Quiroga-Jofre & D. Guerra-Mateo (holotype CBS H-25616, ex-type FMR 20151, CBS 152725).

##### Description.

***Saprobic*** on marine sediments. ***Mycelium*** superficial and immersed, composed of hyaline, septate, branched, smooth-walled, 1.5–2 µm wide hyphae. ***Asexual morph*** with well-differentiated fertile hyphae, arising laterally from vegetative hyphae, branched, straight, sinuous, or contorted, forming intercalary and terminal arthroconidia randomly, 1.5–2 µm wide. ***Arthroconidia*** enteroarthric, 0–1-septate, hyaline, smooth- and thin-walled, cylindrical to subcylindrical or T-shaped, 3.5–4.5(–7.5) × 1.5–2 µm; secession rhexolytic. ***Sexual morph*** heterothallic (observed in FMR 20151 × IHEM 28255 on OA). ***Gymnothecia*** superficial, surrounded by asexual fertile hyphae, single or aggregated, orange-brown, globose to subglobose, 200–310 µm diam. (excluding appendages); peridium composed of a conspicuous network of hyphae, septate, branched, hyaline to brownish orange, finely asperulate, thick-walled, cylindrical, 2.5–4(–4.5) µm wide, with short and long lateral appendages; short appendages arising at acute angles, spine-like, with subacute ends, orange-brown, finely asperulate, 14–25(–30) × 3–4 µm; long appendages arising from peridium at acute and subacute angles, unbranched, straight or curved, cylindrical, progressively tapering terminally, orange-brown, finely asperulate and thick-walled with a basal knuckle-joint towards the base, smooth terminally, with a rounded or subacute curved apex, 200–560 × 3–4 µm long. ***Asci*** 8-spored, evanescent, irregularly disposed, globose to subglobose, 7 × 5–6 µm. ***Ascospores*** unicellular, pale yellow, yellow in mass, reticulate (reticulation regular with smooth polygonal meshes under SEM), thick-walled, subglobose, 3 × 2.5 µm.

##### Culture characteristics

(after 14 days at 25 °C). Colonies on OA reaching 15–20 mm diam., flat, floccose, white (1A1), submerged towards periphery, margin fimbriate; reverse white (1A1). On PDA, 20–22 mm diam., umbonate, cottony, white (1A1), margin entire and diffuse; reverse orange-white (5A2) to white (1A1). On PYE, 25–27 mm diam., convoluted, slightly radially sulcate at periphery, cottony, white (1A1), margin entire; reverse uncolored. Diffusible pigment not observed in any of the media studied.

##### Additional specimens examined.

Belgium, Duffel, on bat fur (*Myotis daubentonii*), October 2018, C. Van den Eynde (IHEM 28255).

##### Cardinal temperatures for growth.

Minimum 5 °C (1 mm), optimum 25–30 °C (22–25 mm), maximum 35 °C (1 mm).

##### Habitat and geographic distribution.

Bat fur and marine sediment. Belgium and Spain.

##### Notes.

*Malbranchea parafilamentosa* represents an independent lineage related to *M. thaxteri* and *M. filamentosa* (Fig. 3). The three species are characterized by the production of fertile hyphae that produce arthroconidia randomly. The morphological differences between their asexual morphs are subtle, but *M. parafilamentosa* shows conspicuously sinuous or contorted fertile hyphae, while in *M. filamentosa*, they are straight and rarely slightly sinuous (Sigler et al. 2002; Rodríguez-Andrade et al. 2021). Their sexual morphs display gymnothecia with short and long appendages, subglobose asci, and reticulate, subglobose ascospores. *Malbranchea parafilamentosa* and *M. filamentosa* are characterized by being heterothallic and producing yellow mass ascospores. These species can be distinguished by the width of the peridial hyphae (2.5–4(–4.5) µm and 4.5–6 µm) and the length of the long peridial appendages (200–560 µm and 100–290 µm) (Sigler et al. 2002). Although the lengths of the peridial appendages overlap between *M. parafilamentosa* and *M. thaxteri* (200–560 µm and 250–540 µm), the latter species is characterized by being homothallic and producing hyaline ascospores (Kuehn 1955; Orr and Kuehn 1971).

Of note is that our phylogenetic analyses show that the strains CBS 198.92 and CBS 146932 (Figs 2, 3), labeled as *Malbranchea setosa* and *Malbranchea stricta*, respectively, are genetically identical to *M. filamentosa* (UAMH 4097) (Sigler et al. 2002). However, the authors who sent the specimen to the CBS collection never formally described *M. setosa*, so this name should be deemed invalid, and *M. stricta* should be considered a synonym.

#### ﻿ Malbranchea sedimenticola

Taxon classificationAnimaliaOnygenalesGymnoascaceae

Guerra-Mateo, Cano & Gené sp. nov.

D566DC64-6B98-5C6D-9A46-80EBCD554333

856497

Fig. 9

##### Etymology.

Latin *sedimentum*, settling, and Latin -*cola*, to inhabit, referring to the species’ preference for soil and sediment substrates.

**Figure 9.**
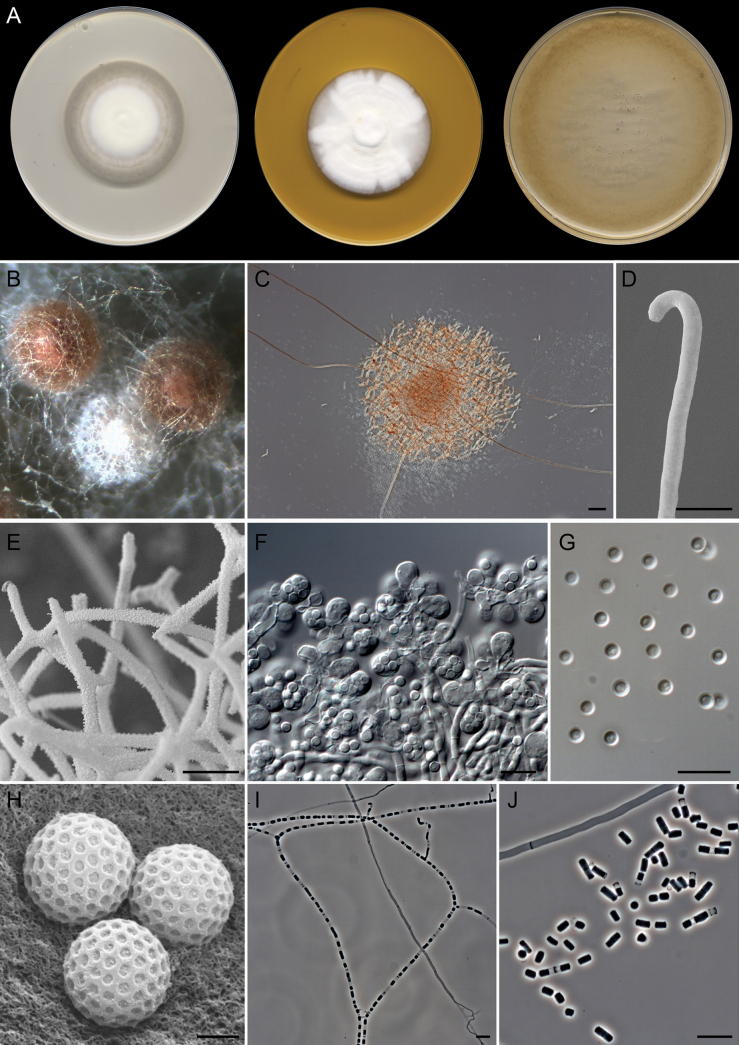
*Malbranchea sedimenticola* (ex-type FMR 19564). A. Colonies on PDA, PYE, and OA after 14 days at 25 °C. B, C. Ascomata. D. Tip of a long appendage under SEM. E. Peridial hyphae under SEM. F, G. Asci and ascospores under light microscopy. H. Ascospore ornamentation under SEM. I, J. Fertile hyphae and arthroconidia. Scale bars: 10 µm (B, C, E–G, I, J); 5 µm (D); 1 µm (H).

##### Type.

Spain • Catalonia, Mediterranean coast, Tarragona, Platja del Miracle, 41°6'6"N, 1°15'44"E, from sediments at 27 m depth, June 2021, G. Quiroga-Jofre & D. Guerra-Mateo (holotype CBS H-25615, ex-type FMR 19564, CBS 152723).

##### Description.

***Saprobic*** on marine sediments. ***Mycelium*** superficial and immersed, composed of septate, branched, hyaline, smooth-walled, 1.5–2.5 µm wide hyphae. ***Asexual morph*** with intercalary or terminal fertile hyphae growing directly from the vegetative hyphae, often anastomosed, straight, slightly branched, branches short and sinuous, forming arthroconidia randomly, 1.5–2.5 µm wide. ***Arthroconidia*** enteroarthric, intercalary or terminal, 0–1-septate, hyaline, smooth- and thin-walled, cylindrical to subcylindrical or T-shaped, (2.5–)4.5–5.5(–7.5) × 1.5–2.5 µm; secession rhexolytic. ***Sexual morph*** homothallic. ***Gymnothecia*** observed on OA, superficial, single or aggregated, orange-brown, globose to subglobose, 350–450 µm diam. (excluding appendages); peridium composed of a conspicuous network of hyphae, septate, branched, hyaline to brownish orange, asperulate, thick-walled, cylindrical, 2.5–3 µm wide, with short and long lateral appendages; short appendages arising at acute angles, spine-like, with subacute to truncated ends, orange-brown, asperulate, 15–40 µm long; long appendages arising from peridium at acute and subacute angles, unbranched, straight or curved, cylindrical, progressively tapering terminally, orange-brown, asperulate and thick-walled toward the base, paler and smooth terminally, with a rounded or subacute curved apex and a basal knuckle-joint, 500–850 µm long. ***Asci*** 8-spored, evanescent, irregularly disposed, globose, subglobose, or pyriform, 7.5–9 × 5.5–6 µm. ***Ascospores*** unicellular, pale yellow, smooth-walled to slightly echinulate (reticulation regular with smooth polygonal meshes under SEM), thick-walled, globose, 2.5–3 µm diam.

##### Culture characteristics

(after 14 days at 25 °C). Colonies on OA reaching 40 mm diam., flat, glabrous at center with abundant ascomata, white (1A1), producing sparse aerial mycelium towards periphery; reverse uncolored. On PDA, 46–50 mm diam., slightly umbonate, cottony, light yellow (4A4) to white at periphery, margin entire and diffuse; reverse reddish orange (7B8) to light orange (5A4), white towards periphery. On PYE, 47 mm diam., slightly umbonate, cottony or glabrous (CBS 319.61), white (1A1), with light yellow patches at center, margin entire and diffuse; reverse uncolored. Diffusible pigment not observed in any of the media studied.

##### Additional specimens examined.

Spain • Catalonia, Mediterranean coast, Tarragona, Platja de la Arrabassada, 41°6'45"N, 1°16'51"E, from sediments at 27 m depth, June 2022, G. Quiroga-Jofre & D. Guerra-Mateo (FMR 20150); • ibid., Barcelona, discharging area of the Llobregat River, 41°17'20´´N, 2°9'16´´E, from sediments at 19 m depth, October 2023, P. Rojas & D. Guerra-Mateo (FMR 21121); the USA, California, Stanislaus Co., on soil, G.F. Orr (CBS 319.61).

##### Cardinal temperatures for growth.

Minimum 5 °C (3 mm), optimum 25 °C (50 mm), maximum 37 °C (17 mm).

##### Habitat and geographic distribution.

Marine sediments and soil in Spain and the USA. In GlobalFungi, in soil from different environments (forest, shrubland, grassland, desert, cropland, and urban), rhizosphere soil, roots, and marine sediment. Australia, Chile, China, Mexico, Morocco, Oman, Qatar, and Tunisia (Fig. 4).

##### Notes.

*Malbranchea sedimenticola* is phylogenetically related to *M. sinuata* and *M. albolutea* (Fig. 3). These species can be distinguished based on colony colour and arthroconidia size. On OA and PYE, *M. sedimenticola* displays white mycelium, while *M. albolutea* produces colonies in shades of pale yellow and *M. sinuata* in shades of yellow to orange and reddish brown. Microscopically, the three species produce the characteristic sinuous fertile hyphae of *Malbranchea* but differ in arthroconidia length, *M. sedimenticola* [(2.5–)4.5–5.5 (–7.5) µm], *M. albolutea* [(1.5–)2–5(–6.5) µm] (Sigler and Carmichael 1976), and *M. sinuata* (1.5–3 µm) (Torres-Garcia et al. 2023). In addition, only *M. sedimenticola* and *M. albolutea* produce the sexual morph, resembling that of the phylogenetically distant species *M. thaxteri*. The ascomatal peridium consists of yellow to brownish-orange, asperulate hyphae that produce short, spine-like appendages and long, straight to uncinate appendages, subglobose asci, and globose ascospores of 2.5–3 µm diam. Although the sexual morphs of *M. sedimenticola* and *M. albolutea* are morphologically similar, they can be distinguished from *M. thaxteri* by showing longer appendages (500–850 µm and 400–800 µm vs. 252–542 µm, respectively) and smooth to slightly echinulate ascospores under a bright field microscope (Sigler and Carmichael 1976), while *M. thaxteri* ascospores are echinulate-reticulate.

It is worth mentioning that the strain CBS 319.61 was formerly identified as *M. thaxteri*, probably based on ascomata morphology. Although this strain remained sterile throughout the culture media assessed in this study, our phylogenetic analyses support the classification of CBS 319.61 as *M. sedimenticola* (Figs 2, 3).

#### ﻿ Malbranchea seminuda

Taxon classificationAnimaliaOnygenalesGymnoascaceae

Guerra-Mateo, Cano & Gené sp. nov.

84078856-EA01-5B2D-80A2-95BA4FC19A09

856496

Fig. 10

##### Etymology.

Latin *semi*-, half, and Latin *nūda*, naked, referring to the ascomata with slightly differentiated peridial hyphae.

**Figure 10.**
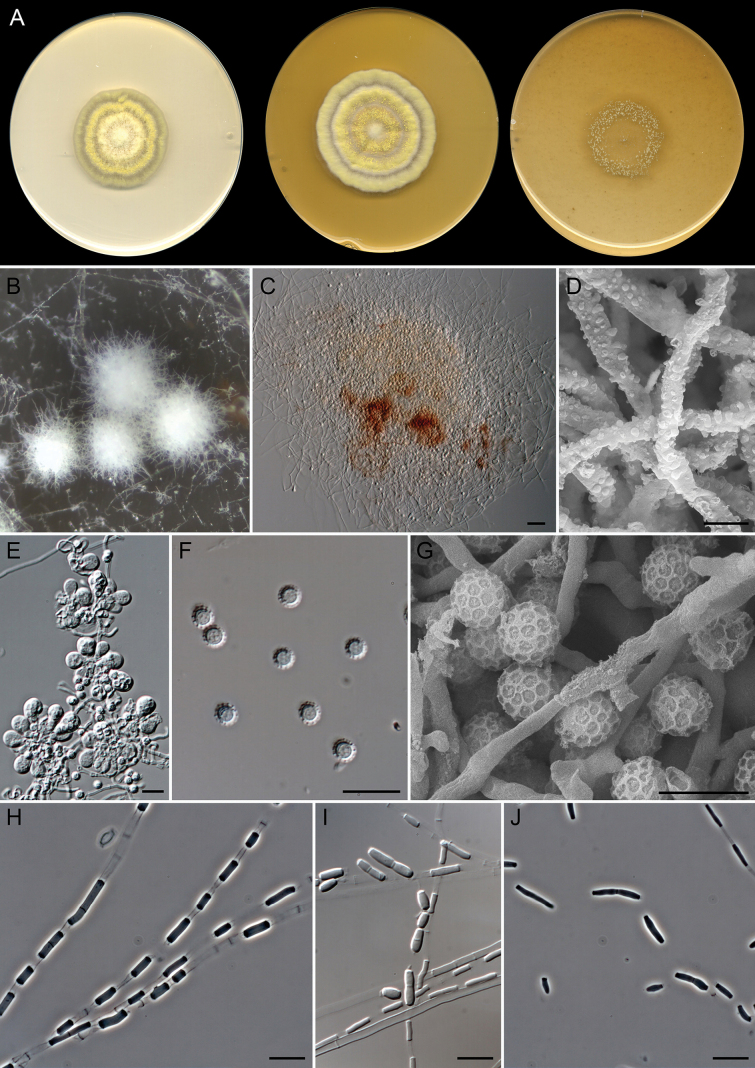
*Malbranchea seminuda* (ex-type FMR 19403). A. Colonies on PDA, PYE, and OA after 14 days at 25 °C. B, C. Ascomata. D. Peridial hyphae ornamentation under SEM. E. Asci. F. Ascospores under light microscopy. G. Ascospore ornamentation under SEM. H–J. Fertile hyphae and arthroconidia. Scale bars: 25 µm (C); 10 µm (E, F, H–J); 5 µm (G); 2.5 µm (D).

##### Type.

Spain • Catalonia, Mediterranean coast, Tarragona, Platja del Miracle, 41°6'6"N, 1°15'44"E, from sediments at 27 m depth, June 2021, G. Quiroga-Jofre & D. Guerra-Mateo (holotype CBS H-25614, ex-type FMR 19403, CBS 152722).

##### Description.

***Saprobic*** from marine environment. ***Mycelium*** superficial and immersed, composed of hyaline, septate, branched, smooth-walled, 2.5–3 µm wide hyphae. ***Asexual morph*** with straight, branched, cylindrical, 2.5–3 µm wide fertile hyphae, giving rise to arthroconidia in a random disposition. ***Arthroconidia*** enteroarthric, 0–1-septate, hyaline, smooth- and thin-walled, cylindrical to subcylindrical or T-shaped, (4–)12–13(–14.5) × 2.5–3 µm; secession rhexolytic. ***Sexual morph*** homothallic. ***Gymnothecia*** observed in all media tested, superficial, aggregated, white to pale yellow, turning pale orange-brown in old cultures (8 weeks), globose to subglobose, 350–500 µm diam.; peridium composed of a subtle network of hyphae, septate, branched, hyaline to pale yellow, verruculose to verrucose (tuberculate under SEM), 2.5–3 µm wide. ***Asci*** 8-spored, evanescent, irregularly disposed, subglobose or pyriform, 11.5–14 × 9–10.5 µm. ***Ascospores*** unicellular, pale yellow, reticulate (reticulation regular with polygonal meshes and conspicuous ridges under SEM), thick-walled, globose, 4–5 µm diam.

##### Culture characteristics

(after 14 days at 25 °C). Colonies on OA reaching 30 mm, flat, with sparse white aerial mycelium at center and glabrous towards periphery, margin diffuse, ascomata abundant and densely aggregated in a concentric ring; reverse uncolored. On PDA, 37 mm diam., slightly floccose, with concentrical rings, greenish yellow (1A6), margin entire and diffuse, ascomata abundant arranged in concentric rings; reverse brownish yellow (5C8) at center, yellowish brown (5E8) and yellowish white (1A2) at periphery. On PYE, 44 mm diam., slightly raised with concentrical rings, radially sulcate, floccose, white (1A1) to greenish yellow (1A6), margin entire, submerged in the medium, ascomata abundant arranged in concentric rings; reverse uncolored. Diffusible pigment not observed in any of the media studied.

##### Cardinal temperatures for growth.

Minimum 5 °C (1 mm), optimum 25 °C (37 mm), maximum 30 °C (24 mm).

##### Habitat and geographic distribution.

Marine sediments in Spain. In GlobalFungi, in soil from different environments (forest, shrubland, and grassland) and marine and terrestrial sediments. Germany, Greece, the Netherlands, South Africa, Spain, the USA, and Zimbabwe (Fig. 4).

##### Notes.

*Malbranchea seminuda* is phylogenetically related to *M. longispora* and *M. multiseptata* (Fig. 3). The three species produce an asexual morph consisting of hyaline and smooth-walled enteroarthric conidia. However, they differ in conidia disposition and size, with *M. longispora* showing straight chains of larger conidia (4–24 × 1–5.5 µm) (Crous et al. 2013), *M. seminuda* displaying straight chains of medium-size conidia (4–)12–13(–14.5) × 2.5–3 µm, and *M. multiseptata* producing straight and sinuous chains of smaller conidia (3–9 × 1.5–2 µm) (Rodríguez-Andrade et al. 2021). In addition, only *M. seminuda* and *M. longispora* produce sexual morphs, which differ in the peridium morphology; while the former species shows a subtle network of hyaline and verruculose to verrucose hyphae, *M. longispora* displays a conspicuous network of pale yellow to orange-brown, thick-walled, tuberculate hyphae (Crous et al. 2013). Other phylogenetically distant species with ascomata morphologically similar to those of *M. seminuda* are *M. concentrica* and *M. kuehnii*. The three species can be distinguished by the ornamentation pattern of the peridial hyphae, being verrucose in *M. seminuda* and *M. kuehnii* but smooth-walled in *M. concentrica* (Solé et al. 2002). *Malbranchea seminuda* differs from *M. kuehnii* in the colour of the ascospores (pale yellow vs. hyaline, respectively) and colonies on PDA (greenish-yellow vs. brown, respectively) (Solé et al. 2002).

#### ﻿ Malbranchea sexualis

Taxon classificationAnimaliaOnygenalesGymnoascaceae

Guerra-Mateo, Cano & Gené sp. nov.

B3D0C136-EC33-524D-B5D5-4E2D8E0E268A

856499

Fig. 11

##### Etymology.

Latin *sexus*, sex, referring to the exclusive presence of the sexual morph in the type strain.

**Figure 11.**
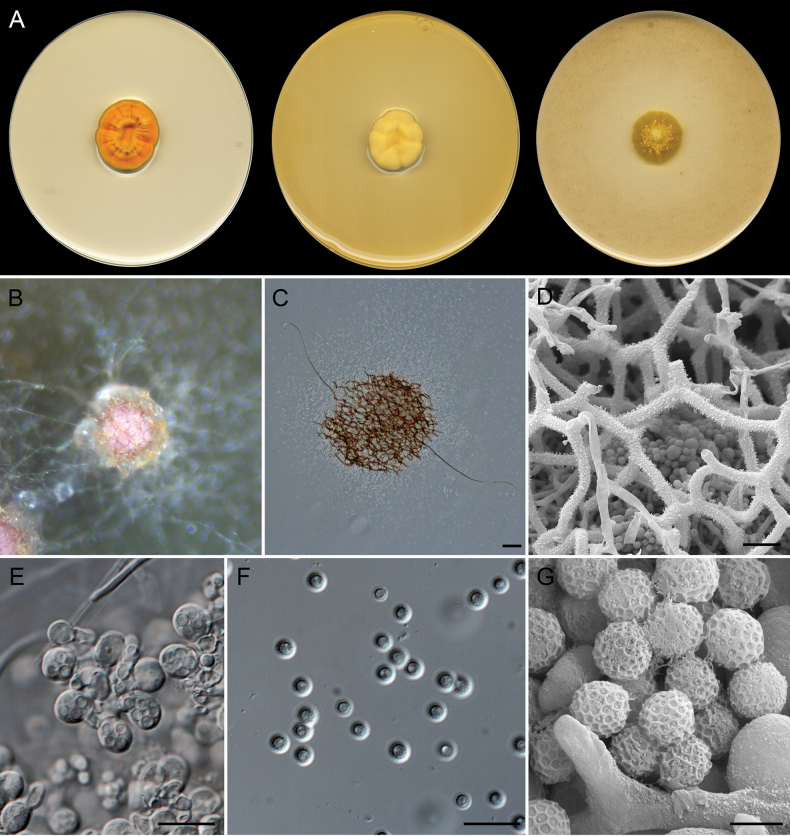
*Malbranchea sexualis* (ex-type FMR 20852). A. Colonies on PDA, PYE, and OA after 14 days at 25 °C. B, C. Ascomata. D. Peridial hyphae ornamentation under SEM. E, F. Asci and ascospores under light microscopy. G. Ascospore ornamentation under SEM. Scale bars: 50 µm (C); 10 µm (D–F); 1 µm (G).

##### Type.

Spain • Catalonia, Mediterranean coast, discharging area of the Ebro River, 40°44'21"N, 0°55'31"E, from sediments at 27 m depth, May 2023, Q. Roca & D. Guerra-Mateo (holotype CBS H-25617, ex-type FMR 20852, CBS 152726).

##### Description.

***Saprobic*** on marine sediments. ***Mycelium*** superficial and immersed, composed of hyaline, septate, branched, smooth-walled, 1.5–2 µm wide hyphae. ***Racket hyphae*** present. ***Asexual morph*** not observed. ***Sexual morph*** homothallic. ***Gymnothecia*** observed on OA, superficial, single or confluent, pale yellow, globose to subglobose, 250–420 µm diam. (excluding appendages); peridium composed of a conspicuous network of hyphae, septate, branched, hyaline to orange-brown, asperulate, thick-walled and cylindrical, 3–4.5 µm wide, with short and long lateral appendages; short appendages arising at acute angles, spine-like, with subacute to rounded ends, orange-brown, finely asperulate, 15–25 µm long; long appendages arising from peridium at acute and subacute angles, unbranched, straight or curved, cylindrical, progressively tapering terminally, orange-brown, asperulate and thick-walled toward the base, paler and smooth terminally, with a rounded or subacute curved apex and mostly with a basal knuckle-joint, 150–340 µm long. ***Asci*** 8-spored, evanescent, irregularly disposed, globose, subglobose, or pyriform, 6–8 × 4.5–5.5 µm. ***Ascospores*** unicellular, pale yellow, smooth-walled to slightly echinulate (reticulation regular with smooth polygonal meshes under SEM), thick-walled, globose, 2–2.5 µm diam.

##### Culture characteristics

(after 14 days at 25 °C). Colonies on OA reaching 20 mm diam., flat with sparse aerial mycelium, yellowish white (4A2) at initial stages, brownish yellow (5C7) when mature, margin entire, ascomata abundant and densely aggregated in center; reverse orange (5B8) to pale orange (5A3). On PDA, 22–26 mm diam., crateriform, radially sulcate at periphery, light orange to orange, velvety, margin entire to slightly lobate; reverse brownish orange (7C8) at center, deep yellow (4A8) towards periphery. On PYE, 26–28 mm diam., elevated, slightly crateriform, radially sulcate, velvety, deep yellow (4A8) at center, light orange (5A5) towards periphery, margin entire, slightly lobate; reverse brownish orange (7C8) to deep yellow (4A8). Diffusible pigment not observed in any of the media studied.

##### Cardinal temperatures for growth.

Minimum 5 °C (1.5 mm), optimum 25 °C (22–26 mm), maximum 30 °C (14 mm).

##### Habitat and geographic distribution.

Marine sediments in Spain. In GlobalFungi, in rhizosphere soil and soil from different environments (forest, woodland, shrubland, grassland, desert, wetland, cropland, and urban), marine sediment, aquatic plant shoots, terrestrial plant shoots and roots. Australia, China, England, Estonia, Finland, Germany, Russia, Spain, Sweden, Tunisia, and the USA (Fig. 4).

##### Notes.

*Malbranchea sexualis* represents an independent lineage phylogenetically related to *M. gymnoascoides*, *M. ostraviensis*, *M. phuphaphetensis*, and *M. umbrina* (Fig. 3). Macroscopically these species are characterized by the production of colonies in shades of yellow and orange, with *M. ostraviensis* being characterized by the production of a reddish diffusible pigment on MEA, PDA, and PCA that has not been observed in the other species. Regarding the reproductive structures, *M. sexualis* is characterized by exclusively producing the sexual morph, while *M. phuphaphetensis* is only known by its asexual morph (Preedanon et al. 2023). On the other hand, *M. gymnoascoides*, *M. ostraviensis*, and *M. umbrina* produce both sexual and asexual morphs. The species that display sexual morph produce gymnothecia with similar features (i.e., yellow to brownish orange, asperulate peridial hyphae with short spine-like appendages and long appendages, globose to pyriform asci, and globose ascospores), but can be distinguished by the length of the peridial appendages and ascospore ornamentation. *Malbranchea umbrina* displays the shortest appendages (5–72 µm) and reticulate ascospores (Hubka et al. 2013), *M. ostraviensis* displays the longest appendages (350–600 µm) and smooth (reticulate with a central depression under SEM) ascospores (Hubka et al. 2013), and both *M. sexualis* and *M. gymnoascoides* produce appendages of overlapping length (150–340 µm vs. 250–400 µm, respectively; Rodríguez-Andrade et al. 2021), but differ in ascospore ornamentation. While the ascospores in *M. gymnoascoides* are smooth-walled (Rodríguez-Andrade et al. 2021), those of *M. sexualis* are smooth to slightly echinulate (reticulate under SEM). Although we have not observed the asexual morph for *M. sexualis* in any of the media tested, the great phylogenetic distance and the morphological characters of the sexual morph confirm it as a novel species.

## ﻿Discussion

This work aimed to characterize the diversity of culturable *Onygenales* from marine sediments along the western Mediterranean coast of Catalonia. Among the 22 species identified, one was determined to be a novel species of *Gymnoascoideus*, four were novel species of *Malbranchea*, and one represented a new genus, *Deilomyces* (Table 1). These taxa were recognized based on established mycological best practices: (1) incorporating strains from multiple collections, (2) integrating morphological, phylogenetic, ecological, and geographic data, and (3) evaluating the species hypothesis within the framework of the consolidated species concept (Quaedvlieg et al. 2014; Aime et al. 2021). In addition, these fungi have proven their ability to grow under osmotic pressure and to degrade cellulose, keratin, and chitin (e.g., *A. crassitunicatus*, *A. fulvescens*, *B. ceratinophila*, *M. parafilamentosa*, *M. sexualis*, *M. sinuata*, *My. keratinophilum*, and *S. casei*). The detection of this onygenalean community in marine sediments suggests that we are only beginning to uncover the role that this environment plays in shaping fungal diversity.

### ﻿Global biogeography of the strains recovered from marine sediments

The geographical distribution of fungi has been a long-standing question in mycology. Advances in DNA-based methods have significantly enhanced our understanding of species boundaries, increasing fungal diversity and challenging the traditional notion that microscopic fungi are ubiquitous (Hawksworth and Lücking 2017; Niskanen et al. 2023). Metabarcoding analyses have determined *Onygenales* as a small but significant component of soils worldwide, with *Onygenaceae* and *Gymnoascaceae* as the most prevalent families (Coleine et al. 2022). Our search in GlobalFungi supports these observations, with all species recovered from marine sediments displaying a strong association with soil and terrestrial habitats. It is noteworthy that, in a similar fashion to soil, *Gymnoascaceae* was the most prevalent and diverse family in marine sediments. Members of *Onygenaceae*, *Neoarthropsidaceae*, and *Malbrancheaceae* were also detected, with the latter as a particularly diverse group (Table 1). Among this diversity, some species displayed a ubiquitous distribution. The species *A. fulvescens*, *G. reessii*, and *My. keratinophilum* represented abundant and widespread fungi in both hemispheres (Suppl. material 2: fig. S18); their frequent detection in culture-dependent surveys emphasizes their high colonization capacity and environmental prevalence (Guarro et al. 2012). The geographical distribution of the remaining species of *Aphanoascus* and *Gymnoascus* detected in marine sediments covered both hemispheres, but their abundance was notably lower. In contrast, the species of *Malbranchea* and *Gymnoascoideus* displayed restricted patterns of distribution.

The genus *Malbranchea* represents one of the most prevalent groups of *Onygenales* in soil (Coleine et al. 2022). The species *M. ostraviensis* and *M. zuffiana* were detected around the globe. It is surprising that these species can be detected abundantly in environmental samples through metabarcoding but are rarely recovered in culture. Indeed, *M. ostraviensis* was described from clinical samples before evidencing its presence in the environment (Hubka et al. 2013). In contrast, other species displayed a restricted distribution, such as *M. seminuda* in areas with Mediterranean climates and *M. sedimenticola* in desertic areas and shrublands (Fig. 4) (Olson et al. 2001). This latter species was able to grow up to 37 °C, supporting this limited range of distribution. In addition, it represents a sister lineage to *M. sinuata*, a species that, despite its original description as a singleton from Spanish freshwater sediments, has been exclusively detected in soil samples from Spain through GlobalFungi (Torres-Garcia et al. 2023). In fact, there are species of *Malbranchea* that are not currently represented in this database, such as *M. parafilamentosa*, *M. echinulata*, and *M. irregularis*, the last two only known from Spanish freshwater sediments (Torres-Garcia et al. 2023). The absence of these species in GlobalFungi suggests their low abundance in nature and their preference for uncommon habitats, including aquatic and animal-associated substrates.

The novel species *Gymnoascoideus alboluteus* represents another uncommon fungal taxon. It was detected in GlobalFungi at low abundance from three forest soil samples originating from Europe, China, and Australia. The considerable geographic distance among these sites suggests a wide distribution range; however, its low abundance may hinder consistent detection. Other species within the genus, such as *Gd. petalosporus* and *Gd. boliviensis*, have been sporadically detected from soil, as well as from human clinical specimens and dung (Orr et al. 1977; Guarro et al. 1992b). These observations support the notion that *Gymnoascoideus* comprises a group of rare environmental fungi.

Conversely, we also identified the opposite scenario: fungi that are abundant in the environment but have never been recovered in culture. Analysis of the ITS2 in GlobalFungi revealed a new lineage, ITS2-ENV1, that is sister to *Gd. alboluteus* (Suppl. material 2: fig. S10) and associated with soil habitats. This lineage is currently known only from sequence data. Such taxa are classified as “dark matter fungi” due to the absence of voucher specimens or cultured isolates, which hampers their formal description (Lücking et al. 2021). The number of dark matter fungi uncovered through metabarcoding is rapidly increasing, driven by both the detection of fungi that are difficult to culture and the exploration of understudied environments, such as aquatic ecosystems (Grossart et al. 2016; Torres-Garcia et al. 2022).

These findings underscore the importance of integrating metabarcoding and culture-based approaches in fungal biodiversity studies. Overall, the GlobalFungi database is a valuable resource for assessing fungal distribution using high-throughput sequencing metabarcoding data. However, it revealed two main limitations: (1) a geographical bias toward samples from the Northern Hemisphere and (2) limited species-level resolution for certain taxa when using only the ITS1 or ITS2 regions (Réblová et al. 2021b). Our exploration of GlobalFungi displayed both widespread taxa and species restricted to specific environmental conditions (Fig. 4; Suppl. material 2: fig. S18). Moreover, ITS1 and ITS2 phylogenetic trees revealed that each region alone was sufficient to differentiate onygenalean species, consistent with prior studies (Moulíková et al. 2023; Torres-Garcia et al. 2023). The only exception, in our case, was the ITS2 region of *Narasimhella*. The species-level resolution of the ITS barcode varies across fungal lineages: while ITS is insufficient for differentiating members of *Chaetomiaceae* and *Podosporaceae*, it remains the primary barcode for identifying *Codinaea*, *Zanclospora*, and most *Onygenales* (Wang et al. 2019, 2022; Réblová et al. 2021a, 2021b; Kandemir et al. 2022; Torres-Garcia et al. 2023). Thus, we conclude that the limitations of GlobalFungi were effectively addressed in our study, and onygenalean fungi are suitable for biogeographical analysis using the ITS region.

### ﻿Culture-dependent detection of *Onygenales* in marine sediments: the effect of technique in diversity surveys

The search in GlobalFungi confirmed the marine environment as an underexplored habitat. The species *A. fulvescens*, *G. reessii*, *Gl. dankaliensis*, and *My. keratinophilum* were associated with marine substrates, particularly in shallow coastal areas (Suppl. material 2: figs S18, S19). Previous surveys in shallow marine sediments from the coasts of Catalonia (western Mediterranean Sea) explored the efficacy of cycloheximide and the hair-baiting method to recover onygenalean fungi in culture (Ulfig et al. 1997, 1998). The hair-baiting method selected fungi that degrade keratin, such as *A. fulvescens* and *A. keratinophilus* (Ulfig et al. 1997). On the other hand, the use of cycloheximide in culture media revealed a broader diversity of onygenalean fungi, with species such as *A. fulvescens*, *Gl. dankaliensis*, *Gl. littoralis*, *M. pulchella*, *M. conjugata*, *M. reticulata*, *M. umbrina*, *M. zuffiana*, *My. keratinophilum*, *N. hyalinospora*, and *N. poonensis* (Ulfig et al. 1998). In this work, we detected most of these species using two isolation methods and four different culture media. Interestingly, most of the species were recovered in culture from sediments at 27 m of depth through the direct plating of the sediment and using cycloheximide (PDA+C) (Table 1). The addition of cycloheximide played a crucial role in unraveling the diversity of onygenalean fungi in marine sediments. This protein-synthesis inhibitor prevented the growth of most environmental fungi, facilitating the detection of these ascomycetes (Wingfield et al. 2022). In fact, most of the strains representing novel species were isolated with PDA+C, independently of the isolation method, direct plating or SMF (Table 1). However, SMF enabled the detection of singleton rare species such as *D. minimus* and *M. seminuda*. The medium PDA+C has also been used to recover *Onygenales* from freshwater sediments of the rivers Ter and Llobregat (Torres-Garcia et al. 2023). These rivers were characterized by species of *Malbranchea* and *Myriodontium*, with *M. ostraviensis* and *My. keratinophilum* as the only species that we also detected in marine sediments. The detection of a different community while using the same isolation medium suggests an underlying process of adaptation to different environmental conditions.

Regarding the other culture media used for isolation, we detected *A. fulvescens* at 27 m of depth using SWMEA3% and demonstrated its ability to colonize human hair (Tables 1, 2). In addition, we detected an additional keratinophilous species, *B. ceratinophila*, at 13 m of depth using DRBC. The detection of this species was surprising because it had not been recovered in culture since its original description from garden soil (Guarro et al. 1987).

Our results emphasize the importance of employing various culture media in diversity surveys. Although onygenalean fungi tend to specialize in keratin degradation, many lineages display different nutritional preferences (Muñoz et al. 2018; Kandemir et al. 2022). In this way, cycloheximide outperforms the hair-baiting method in detecting *Onygenales* diversity in marine sediments. In this sense, seawater-based media (SWMEA3% and SWOA) exhibited a similar effect to PDA+C, facilitating the detection of halotolerant species over Other fungi.

### ﻿*Onygenales* adaptability to the marine environment

The *Onygenales* can be divided into two main groups based on nutrition preferences: those degrading cellulose, which is considered an ancestral trait, and those that have specialized in keratin degradation (Kandemir et al. 2022). During *Onygenales* evolution, cellulases were reduced in favor of keratinases, enabling the colonization of animal-associated substrates (Muñoz et al. 2018). However, some onygenalean fungi can still produce both groups of enzymes. For example, *M. zuffiana* can be recovered as an environmental fungus but is also associated with animal and human clinical specimens (Rodríguez-Andrade et al. 2021). This species is known to predominantly produce keratinases but still maintains cellulolytic domains encoded in its genome (Granados-Casas et al. 2024). Other species that we have detected in marine sediments, like *Gl. dankaliensis* and *M. ostraviensis*, also represent common environmental fungi and causal agents of onychomycosis in humans (Fig. 4, Suppl. material 2: figs S18, S19) (Hubka et al. 2013; de Hoog et al. 2020; Rodríguez-Andrade et al. 2021). In the current study, these species only displayed cellulolytic activity in culture (Table 2), which might be related to the environmental origin of these strains. However, cellulolytic activity and hair degradation were only observed in *A. crassitunicatus*, *A. fulvescens*, and *B. ceratinophila*, all members of *Onygenaceae* (Table 2). This family also includes *Coccidioides*, one of the most relevant genera of dimorphic fungi responsible for systemic infections in humans (de Hoog et al. 2020; Kandemir et al. 2022). The ability of members of this family to degrade both cellulose and keratin, along with their presence in the environment, supports previous findings in clinical mycology suggesting that the environment may serve as a reservoir for clinically relevant fungi (Luberto et al. 2024).

Aquatic environments are characterized by the dominance of chitin as the main polysaccharide (Souza et al. 2011). Chitin is massively produced in the water column but barely detected in sediments, suggesting its degradation (Poulicek and Jeuniaux 1988; Souza et al. 2011). At the same time, aquatic plants and macroalgae represent a source of cellulose (Singhania et al. 2025). It is noteworthy that cellulose and chitin share similar chemical structures; therefore, chitin degradation may follow a similar pathway to cellulose degradation (Wörner and Pester 2019). All the species detected here degraded cellulose, and, in addition, we detected four species with chitinolytic activity from marine sediments: *M. parafilamentosa*, *M. sexualis*, *My. keratinophilum*, and *S. casei*; and one species from freshwater sediments, *M. sinuata* (Table 2). Although fungi are known to produce chitinases for maintaining their cell wall, the annotation of the genome of *M. zuffiana* revealed a great abundance of the GH18 enzyme family, which includes several exogenous chitinases (Granados-Casas et al. 2024; Paudel et al. 2025). The active degradation of chitin by bacteria has been demonstrated in both freshwater and marine sediments (Wörner and Pester 2019). Therefore, onygenalean fungi could play a similar role in these environments.

Salinity represents a physiological barrier to the colonization of the marine environment. Halotolerance has been a major driver of evolution across the eukaryotic tree of life, with multiple transitions between marine and non-marine lineages (Jamy et al. 2022). In fungi, halotolerance represents a common trait among terrestrial lineages, particularly within families like *Gymnoascaceae* (Gunde-Cimerman et al. 2009; Kandemir et al. 2022). This family was the most abundant and diverse in marine sediments (Table 1). Species of *Gymnoascus* and *Narasimhella* grew equally well in simple media and media supplemented with 3.5% salt, but species of *Gymnascella* grew better in media supplemented with 3.5% salt. Most of the members of *Malbrancheaceae* and *Onygenaceae* also tolerated up to 10% NaCl, with the exception of *D. minimus* and *M. seminuda*, which evaded growing on salty media (Table 2). This suggests that those species that tolerated salt could represent metabolically active fungi. In fact, there is an onygenalean species that has been exclusively recovered from the marine environment, *Gl. littoralis* (Landy and Jones 2006; Jones et al. 2019; Kandemir et al. 2022). Although the physiology of marine *Onygenales* remains obscure, it is noteworthy that, in this work, most of the species that degraded chitin, i.e., *M. sinuata*, *M. parafilamentosa*, *M. sexualis*, *My. keratinophilum*, and *S. casei*, also tolerated salt concentrations above marine-like conditions (Table 2).

The diversity of *Onygenales* that we recovered from marine sediments is composed of fungi predominantly associated with terrestrial habitats (Fig. 4, Suppl. material 2: figs S18, S19). Fungal propagules are known to remain viable for extended periods and can be dispersed through the air from terrestrial to marine environments, where they seem to accumulate in sediments (Fröhlich-Nowoisky et al. 2012). Despite the terrestrial origin of this community, the observed halotolerance in most species suggests the ability to adapt metabolically to marine conditions. For example, fungal spores from the Dead Sea have been reported to tolerate higher osmotic pressure than their terrestrial counterparts, suggesting a selection for osmotolerant propagules (Kis-Papo et al. 2003). Structural cell wall rearrangements have been reported in fungi recovered from the marine environment, enabling adaptation to both hypersaline and salt-deprived conditions and providing a mechanism for dealing with external stress (Fernando et al. 2023). Transition events between terrestrial and freshwater habitats to the marine environment seem to have occurred more frequently within fungi than in any other microbial eukaryotes (Jamy et al. 2022; Cunliffe 2023). Thus, the community detected here could represent fungi with the metabolic capacity to survive in both terrestrial and aquatic environments.

### ﻿Species delimitation and phylogeny

The ecological interpretation of the species detected in marine sediments is dependent on the taxonomic analyses developed for identification. For this reason, we identified the strains at the species level, combining morphology and phylogenetic analyses. The order *Onygenales* has primarily been studied using the ITS region since the advent of molecular methods (Untereiner et al. 2004; Arunmozhi Balajee et al. 2007). While this region has been effective in distinguishing species, many group boundaries remained unresolved. In response, the mycological community has made efforts to incorporate protein-coding regions such as *tub2*, *rpb2*, *tef1*, and *actin* into phylogenetic analyses (Kandemir et al. 2022). Unfortunately, there are few public sequences available for some of these genetic markers. In this study, we examined the relationships of marine sediment strains using both ITS and LSU regions (Fig. 2). In addition, we used the *tub2* marker to clarify the phylogenies of *Gymnoascus* (Suppl. material 2: fig. S4) and *Malbranchea* (Fig. 3), strengthening the backbone support of the phylogenies and resolving relationships among sister taxa. There are *rpb2* sequences available for several species of *Malbranchea*. However, their low number hindered a concatenated phylogenetic analysis, and we could only use them to compare percentages of identity.

The morphological and phylogenetic analyses of *Malbranchea* revealed the lack of robust morphological features between species, making morphology-based identifications challenging. Species such as *M. albolutea* and *M. sedimenticola*, which represented sister taxa (Clade II, Fig. 3), exhibited the characteristic features of the genus, including an Auxarthron-like sexual morph with peridial appendages and a sinuous arthroconidial asexual morph. These species were also nearly identical to *M. thaxteri*, a phylogenetically distant species (Clade I, Fig. 3). Meanwhile, species like *M. seminuda*, *M. concentrica*, and *M. kuehnii* (Clade III, Fig. 3) displayed different ascomata features, consisting of subtle peridial hyphae lacking appendages—a morphology that resembles the genus *Amauroascus* rather than *Malbranchea* (Solé et al. 2002). Nevertheless, this clade also included species with Auxarthron-like sexual morphs, such as *M. longispora*. Despite these morphological variations, our phylogenetic analysis (Figs 3, 4), as well as those from other recent studies, supported *Malbranchea* as a monophyletic genus (Kandemir et al. 2022; Torres-Garcia et al. 2023). Given these findings, we attempted to assess speciation events within *Malbranchea*, but unfortunately, posterior probabilities indicated that our data were insufficient for these analyses (Suppl. material 2: fig. S5). Since the recent re-erection of *Malbranchea*, there has been a lot of attention on this genus, and up to seven novel species have been described from freshwater sediments, cave soil, and clinical samples (Rodríguez-Andrade et al. 2021; Preedanon et al. 2023; Torres-Garcia et al. 2023). In this work, we proposed four additional species (Table 2). This increase in diversity suggests that the exploration of uncommon environments, in particular aquatic habitats, may reveal more *Malbranchea* species. Future descriptions of novel taxa in this genus should include protein-coding regions in the phylogenies, at least the *tub2* region, to provide phylogenetic support to the different clades.

The genus *Gymnoascoideus* represents a poorly represented group in phylogenetic analyses of *Onygenales* (Kandemir et al. 2022). Although the type species, *Gd. petalosporus*, has been abundantly recovered in culture, only a couple of strains have been sequenced (Fig. 2). Since the description of *Gymnoascoideus*, only one additional species has been added to the genus, *Gd. boliviensis* (Kandemir et al. 2022). In this work, we introduced a third species, *Gd. alboluteus*, which seems to represent a rare environmental fungus (Fig. 4).

We also introduced the novel genus *Deilomyces*. According to our phylogenetic analysis, *D. minimus* and *Di. rosea* represented two distinct and distant genera placed in a well-supported lineage that remains unresolved at the family level (Fig. 2) (Kandemir et al. 2022). *Diploospora rosea* represents an ecologically rare fungus associated with cardboard and parchments that has only been recovered three times in culture (Tanney et al. 2015). In addition, it displays a mixture of thallic and blastic conidiogenesis with conidia arising in acropetal chains—a reproductive strategy distinct from the Chrysosporium-like asexual morph observed in *Deilomyces*. The great diversity within this small clade highlights the significant gaps in our understanding of certain onygenalean groups and emphasizes the importance of ongoing surveys to explore fungal diversity in environmental substrates.

## ﻿Conclusion

Marine sediments of the western Mediterranean Sea serve as reservoirs of rare and novel onygenalean fungi. The combination of four different culture media proved effective for recovering *Onygenales*, with PDA+C being the most successful for isolating these fungi. While most of the recovered species are common terrestrial inhabitants, others, such as the new species *Gd. alboluteus* and *M. parafilamentosa*, represent rare environmental taxa. We understand this community consists of terrestrial propagules that have reached the marine environment. Notably, species capable of tolerating marine-like salinity and degrading both cellulose and chitin, such as *M. parafilamentosa*, *M. sexualis*, *My. keratinophilum*, and *S. casei*, may be considered facultative marine fungi. However, further metabolic studies are needed to validate this hypothesis. Overall, our findings highlight the high diversity of onygenalean fungi in marine sediments and their remarkable metabolic plasticity. Moreover, the detection of species exhibiting exogenous chitinolytic activity highlights the value of culture-dependent approaches and provides a foundation for future studies on *Onygenales* metabolism and adaptability.

## Supplementary Material

XML Treatment for Deilomyces

XML Treatment for Deilomyces minimus

XML Treatment for Gymnoascoideus alboluteus

XML Treatment for Malbranchea parafilamentosa

XML Treatment for Malbranchea sedimenticola

XML Treatment for Malbranchea seminuda

XML Treatment for Malbranchea sexualis

## ﻿Additional information

### Use of AI

No use of AI was reported.

### Adherence to national and international regulations

All the fungal strains used in this study have been legally obtained, respecting the Convention on Biological Diversity (Rio Convention).

### Author ORCIDs

Daniel Guerra-Mateo https://orcid.org/0000-0002-6168-6179

Josepa Gené https://orcid.org/0000-0001-6195-9299

Pierre Becker https://orcid.org/0000-0001-6158-102X

José F. Cano-Lira https://orcid.org/0000-0003-4495-4394

### Data availability

All of the data that support the findings of this study are available in the main text or Supplementary Information.

## Supplementary materials

Supplementary material 1 Additional information Data type xlsx https://binary.pensoft.net/file/1409057This dataset is made available under the Open Database License (http://opendatacommons.org/licenses/odbl/1.0/). The Open Database License (ODbL) is a license agreement intended to allow users to freely share, modify, and use this Dataset while maintaining this same freedom for others, provided that the original source and author(s) are credited.Daniel Guerra-Mateo, Josepa Gené, Pierre Becker, José F. Cano-Lira

Supplementary material 2 Contains 19 auxiliary figures Data type pdf https://binary.pensoft.net/file/1409058This dataset is made available under the Open Database License (http://opendatacommons.org/licenses/odbl/1.0/). The Open Database License (ODbL) is a license agreement intended to allow users to freely share, modify, and use this Dataset while maintaining this same freedom for others, provided that the original source and author(s) are credited.Daniel Guerra-Mateo, Josepa Gené, Pierre Becker, José F. Cano-Lira
