# Supplementary material for: ﻿ Onygenales from marine sediments: diversity, novel taxa, global distribution, and adaptability to the marine environment
Source: IMA Fungus. 2025 Sep 4;16:e158470. doi: 10.3897/imafungus.16.158470 (PMC12457909; doi:10.3897/imafungus.16.158470)
Supplement: Supplementary material 2 — Contains 19 auxiliary figures [file imafungus-16-e158470-s002.pdf]

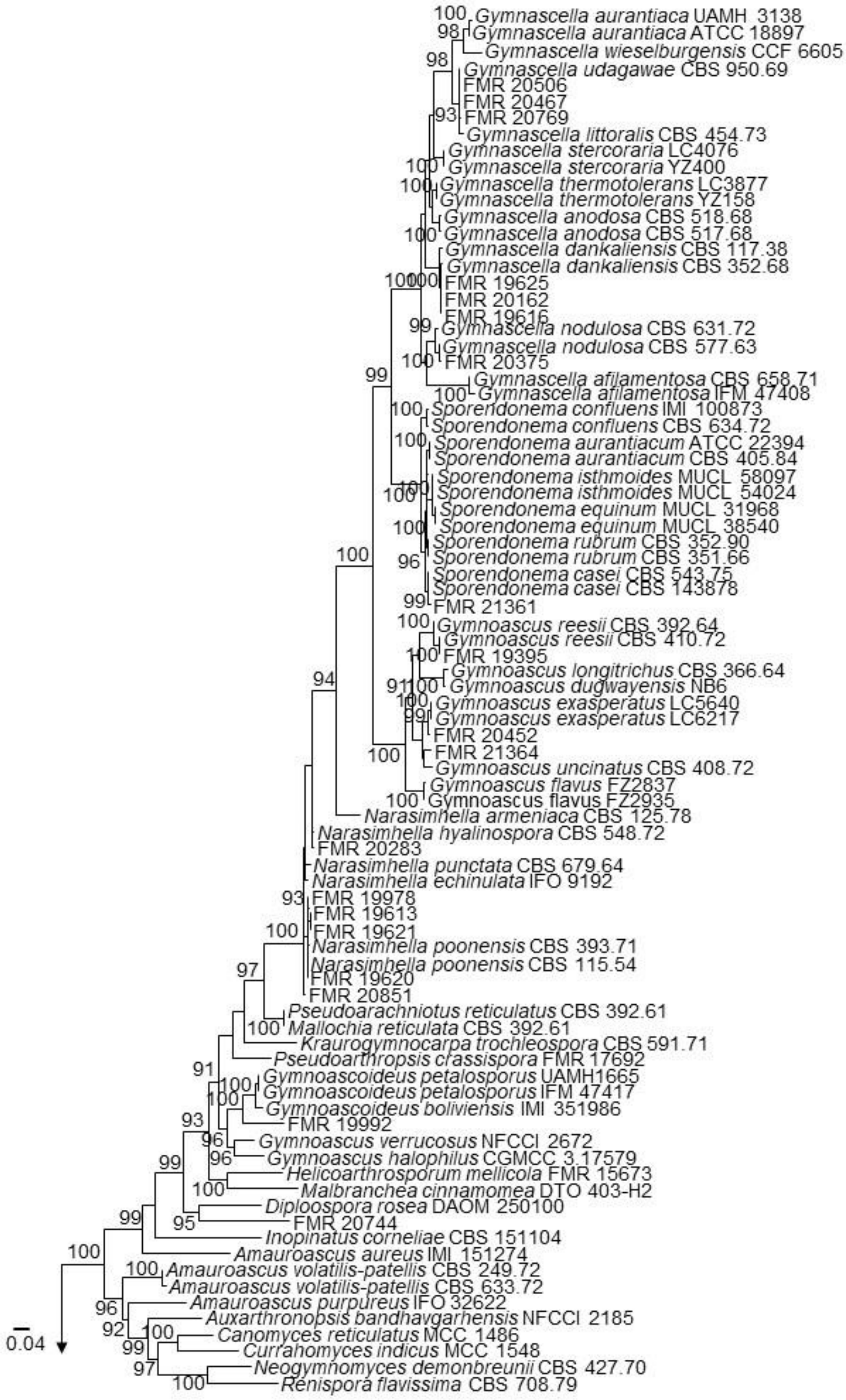
Gymnascella aurantiaca UAMH 3138
Gymnascella aurantiaca ATCC 18897
Gymnascella wieselburgensis CCF 6605
Gymnascella udagawae CBS 950.69
FMR 20506
FMR 20467
FMR 20769
Gymnascella littoralis CBS 454.73
Gymnascella stercoraria LC4076
Gymnascella stercoraria YZ400
Gymnascella thermotolerans LC3877
Gymnascella thermotolerans YZ158
Gymnascella anodosa CBS 518.68
Gymnascella anodosa CBS 517.68
Gymnascella dankaliensis CBS 117.38
Gymnascella dankaliensis CBS 352.68
FMR 19625
FMR 20162
FMR 19616
Gymnascella nodulosa CBS 631.72
Gymnascella nodulosa CBS 577.63
FMR 20375
Gymnascella afilamentosa CBS 658.71
Gymnascella afilamentosa IFM 47408
Sporendonema confluens IMI 100873
Sporendonema confluens CBS 634.72
Sporendonema aurantiacum ATCC 22394
Sporendonema aurantiacum CBS 405.84
Sporendonema isthmoides MUCL 58097
Sporendonema isthmoides MUCL 54024
Sporendonema equinum MUCL 31968
Sporendonema equinum MUCL 38540
Sporendonema rubrum CBS 352.90
Sporendonema rubrum CBS 351.66
Sporendonema casei CBS 543.75
Sporendonema casei CBS 143878
FMR 21361
Gymnoascus reesii CBS 392.64
Gymnoascus reesii CBS 410.72
FMR 19395
Gymnoascus longitrichus CBS 366.64
Gymnoascus dugwayensis NB6
Gymnoascus exasperatus LC5640
Gymnoascus exasperatus LC6217
FMR 20452
FMR 21364
Gymnoascus uncinatus CBS 408.72
Gymnoascus flavus FZ2837
Gymnoascus flavus FZ2935
Narasimhella armeniaca CBS 125.78
Narasimhella hyalinospora CBS 548.72
FMR 20283
Narasimhella punctata CBS 679.64
Narasimhella echinulata IFO 9192
FMR 19978
FMR 19613
FMR 19621
Narasimhella poonensis CBS 393.71
Narasimhella poonensis CBS 115.54
FMR 19620
FMR 20851
Pseudoarachniotus reticulatus CBS 392.61
Mallochia reticulata CBS 392.61
Kraurogymnocarpa trochleospora CBS 591.71
Pseudoarthropsis crassispora FMR 17692
Gymnoascoideus petalosporus UAMH1665
Gymnoascoideus petalosporus IFM 47417
Gymnoascoideus boliviensis IMI 351986
FMR 19992
Gymnoascus verrucosus NFCCI 2672
Gymnoascus halophilus CGMCC 3.17579
Helicoarthrosporum mellicola FMR 15673
Malbranchea cinnamomea DTO 403-H2
Diploospora rosea DAOM 250100
FMR 20744
Inopinatus corneliae CBS 151104
Amauroascus aureus IMI 151274
Amauroascus volatilis-patellis CBS 249.72
Amauroascus volatilis-patellis CBS 633.72
Amauroascus purpureus IFO 32622
Auxarthronopsis bandhavgarhensis NFCCI 2185
Canomyces reticulatus MCC 1486
Currahomyces indicus MCC 1548
Neogymnomyces demonbreunii CBS 427.70
Renispora flavissima CBS 708.79
0.04

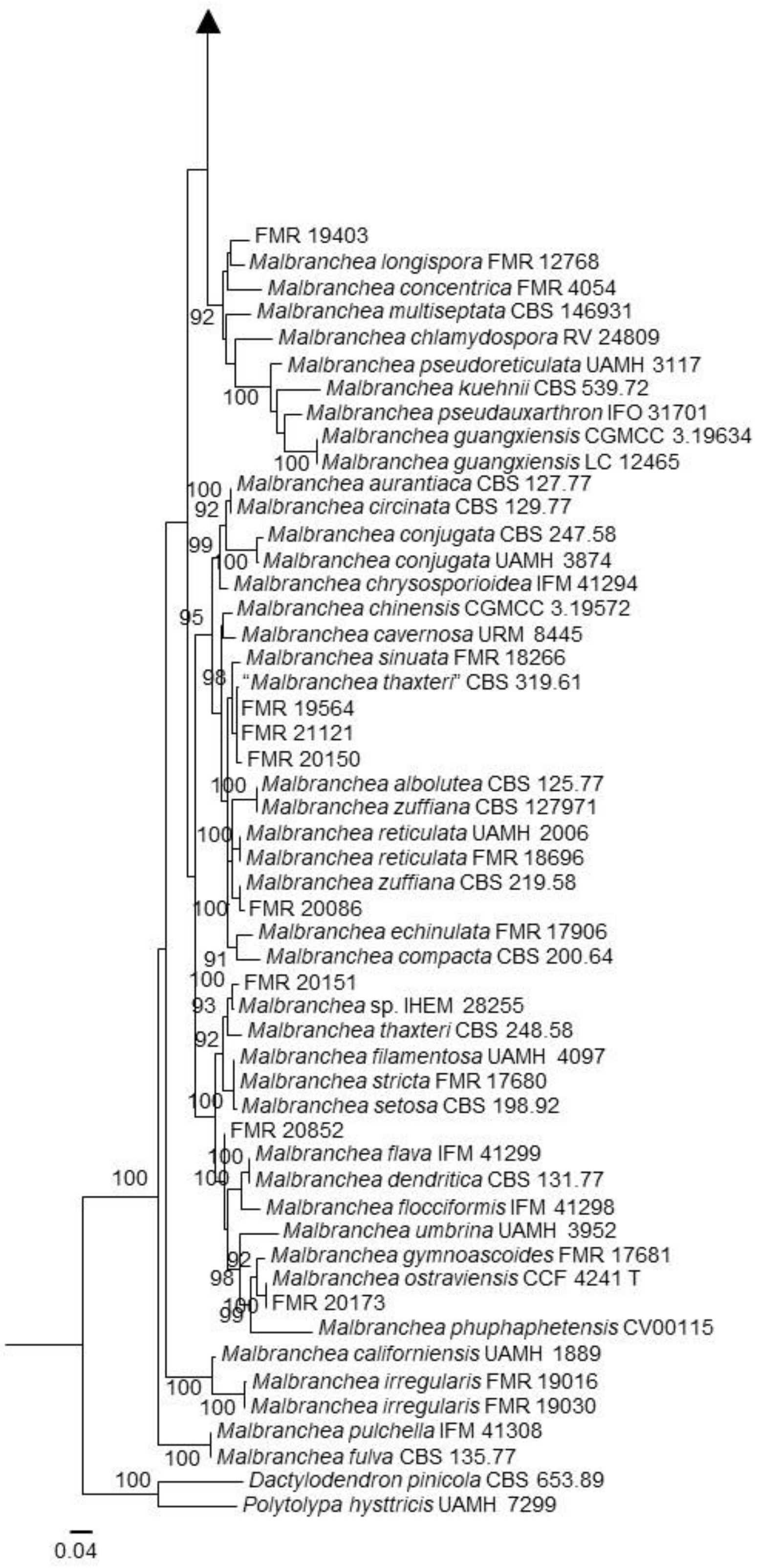

Figure S1 Maximum likelihood tree representing the individual ITS alignment of the families *Gymnoascaceae*, *Malbrancheaceae*, *Neogymnomycetaceae*, and a related *incertae sedis* clade in *Onygenales*.

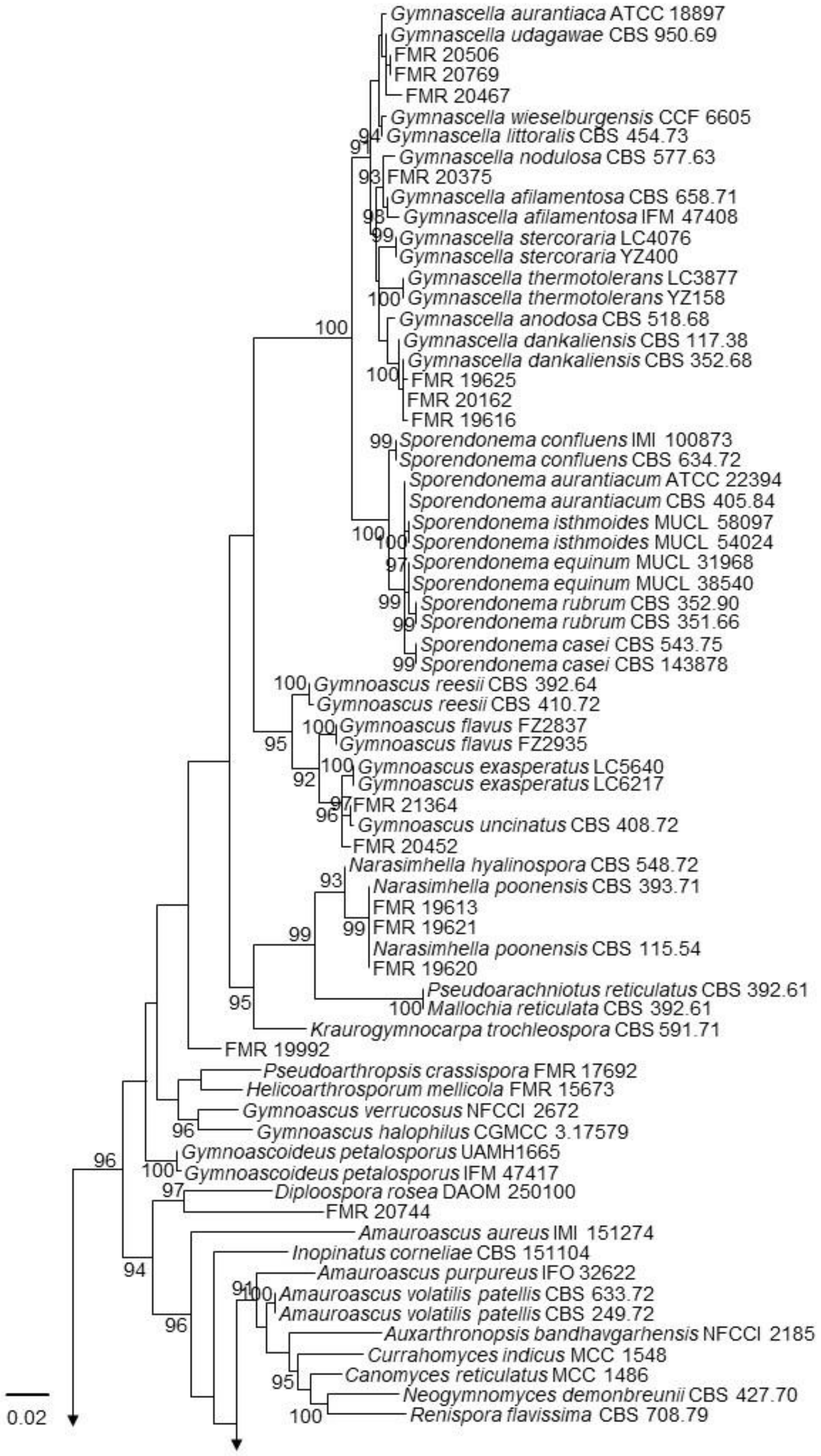

Gymnascella aurantiaca ATCC 18897
Gymnascella udagawae CBS 950.69
FMR 20506
FMR 20769
FMR 20467
Gymnascella wieselburgensis CCF 6605
Gymnascella littoralis CBS 454.73
Gymnascella nodulosa CBS 577.63
FMR 20375
Gymnascella afilamentosa CBS 658.71
Gymnascella afilamentosa IFM 47408
Gymnascella stercoraria LC4076
Gymnascella stercoraria YZ400
Gymnascella thermotolerans LC3877
Gymnascella thermotolerans YZ158
Gymnascella anodosa CBS 518.68
Gymnascella dankaliensis CBS 117.38
Gymnascella dankaliensis CBS 352.68
FMR 19625
FMR 20162
FMR 19616
Sporendonema confluens IMI 100873
Sporendonema confluens CBS 634.72
Sporendonema aurantiacum ATCC 22394
Sporendonema aurantiacum CBS 405.84
Sporendonema isthmoides MUCL 58097
Sporendonema isthmoides MUCL 54024
Sporendonema equinum MUCL 31968
Sporendonema equinum MUCL 38540
Sporendonema rubrum CBS 352.90
Sporendonema rubrum CBS 351.66
Sporendonema casei CBS 543.75
Sporendonema casei CBS 143878
Gymnoascus reesii CBS 392.64
Gymnoascus reesii CBS 410.72
Gymnoascus flavus FZ2837
Gymnoascus flavus FZ2935
Gymnoascus exasperatus LC5640
Gymnoascus exasperatus LC6217
FMR 21364
Gymnoascus uncinatus CBS 408.72
FMR 20452
Narasimhella hyalinospora CBS 548.72
Narasimhella poonensis CBS 393.71
FMR 19613
FMR 19621
Narasimhella poonensis CBS 115.54
FMR 19620
Pseudoarachniotus reticulatus CBS 392.61
Mallochia reticulata CBS 392.61
Kraurogymnocarpa trochleospora CBS 591.71
FMR 19992
Pseudoarthropsis crassispora FMR 17692
Helicoarthrosporum mellicola FMR 15673
Gymnoascus verrucosus NFCCI 2672
Gymnoascus halophilus CGMCC 3.17579
Gymnoascoideus petalosporus UAMH1665
Gymnoascoideus petalosporus IFM 47417
Diploospora rosea DAOM 250100
FMR 20744
Amauroascus aureus IMI 151274
Inopinatus corneliae CBS 151104
Amauroascus purpureus IFO 32622
Amauroascus volatilis patellis CBS 633.72
Amauroascus volatilis patellis CBS 249.72
Auxarthronopsis bandhavgarhensis NFCCI 2185
Currahomyces indicus MCC 1548
Canomyces reticulatus MCC 1486
Neogymnomyces demonbreunii CBS 427.70
Renispora flavissima CBS 708.79
0.02

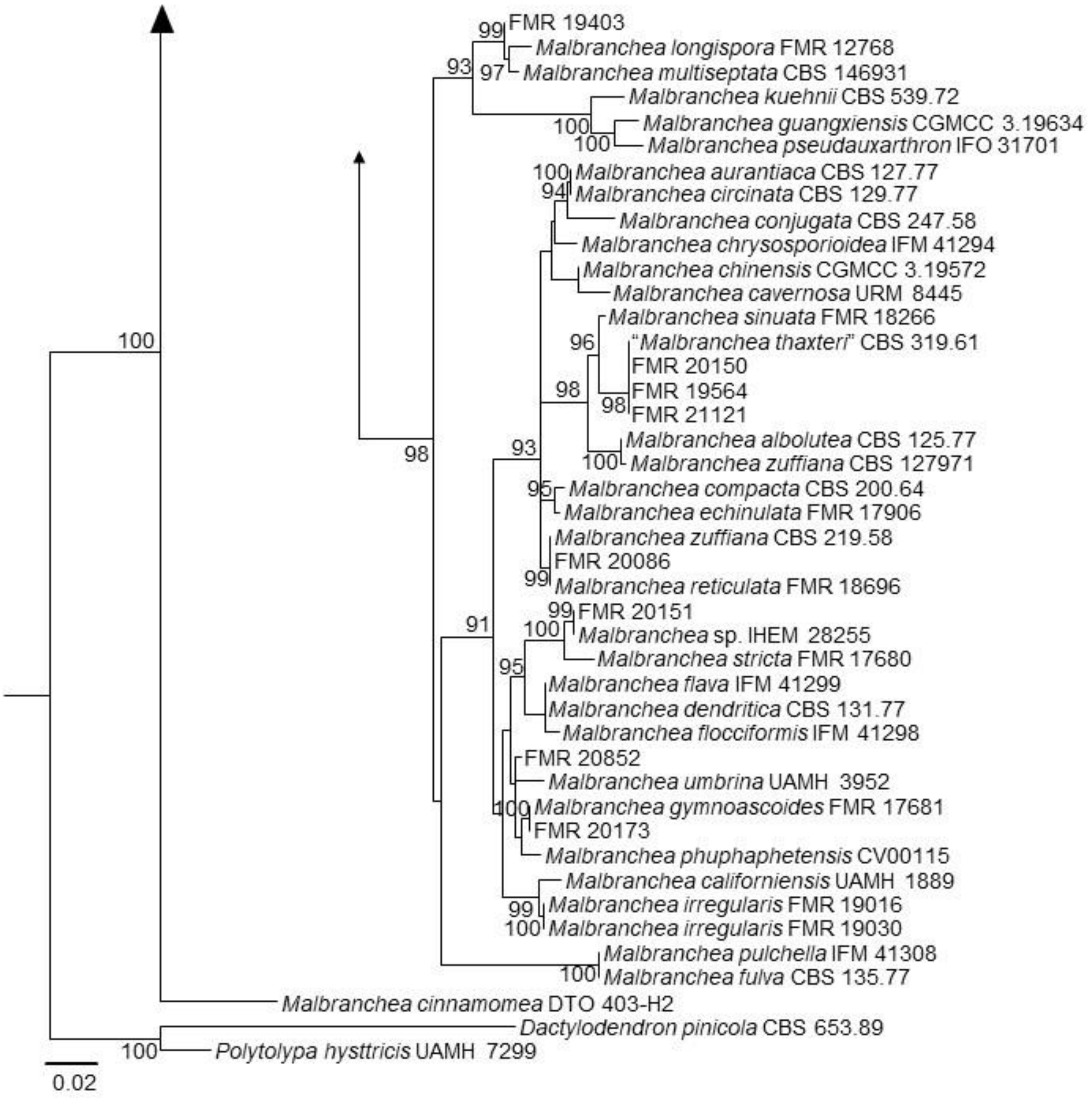

Figure S2 Maximum likelihood tree representing the individual LSU alignment of the families *Gymnoascaceae*, *Malbrancheaceae*, *Neogymnomycetaceae*, and a related *incertae sedis* clade in *Onygenales*.

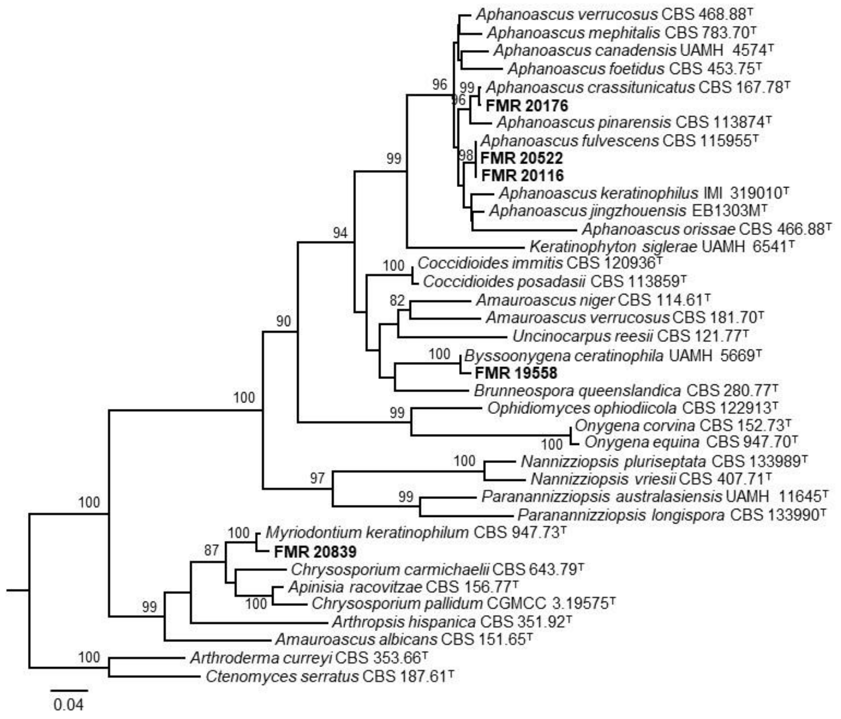

Figure S3 Maximum likelihood tree representing a concatenated ITS-LSU alignment of representative species in *Onygenaceae* and *Neoarthropsidaceae*.

.

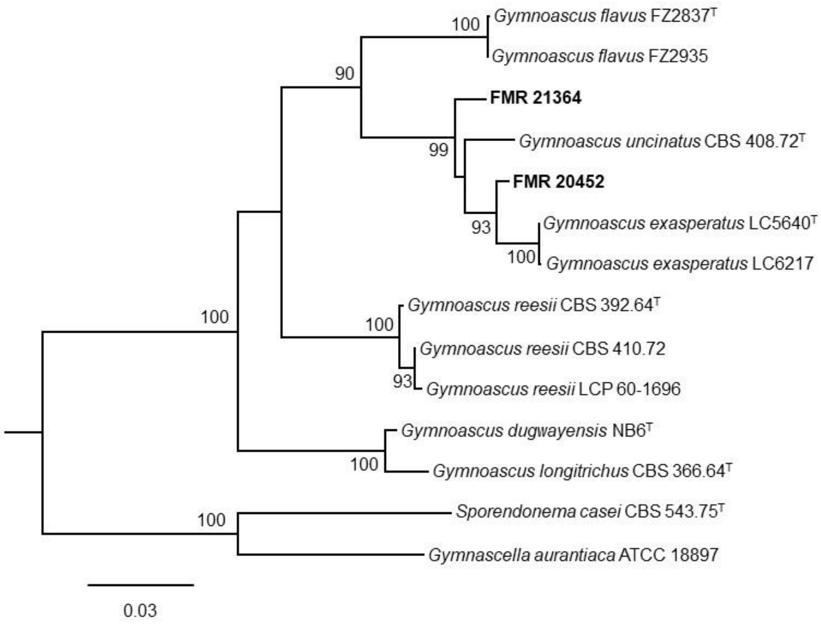

Figure S4 Maximum likelihood tree representing a concatenated ITS-LSU-*tub*2 alignment of *Gymnoascus*.

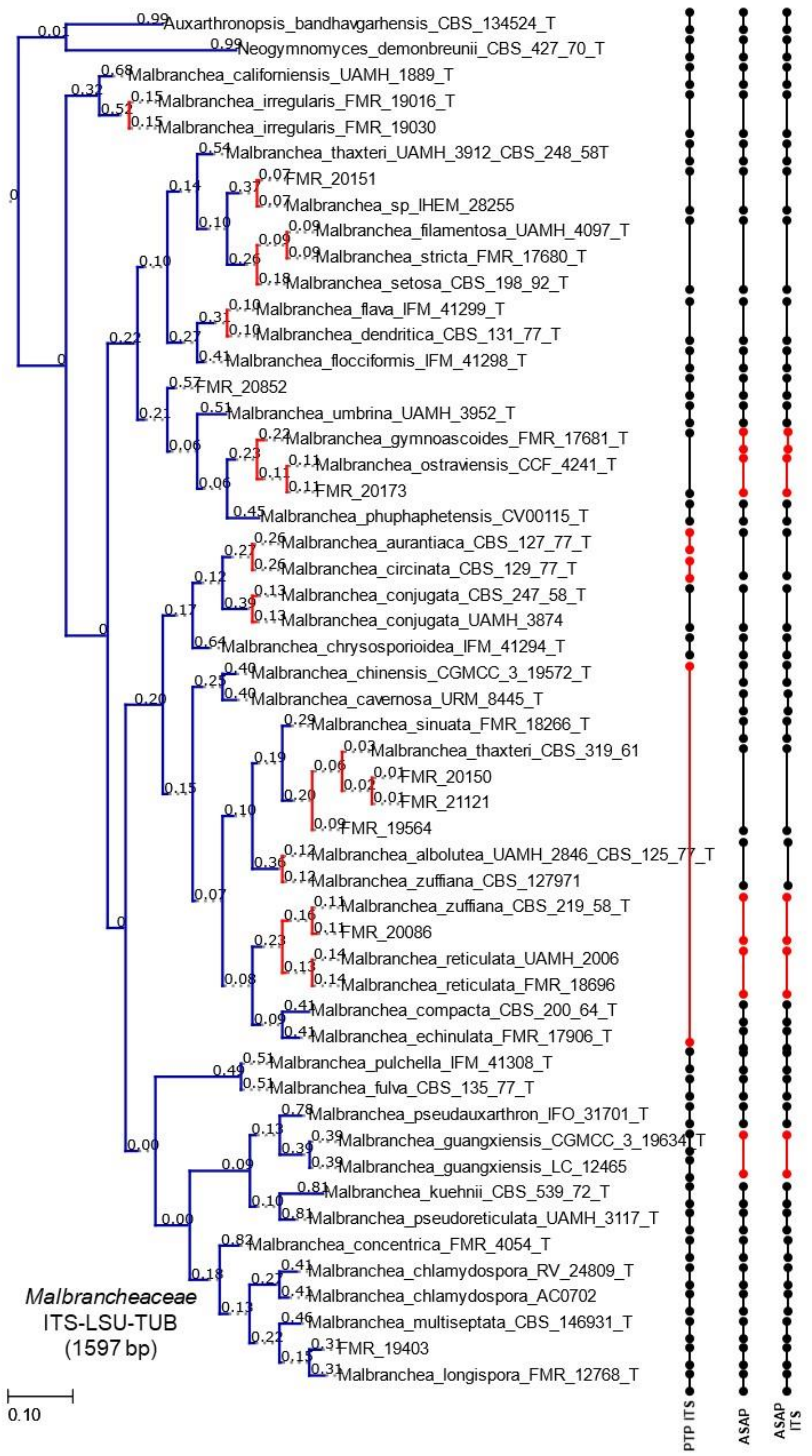

Auxarthronopsis_bandhavgarhensis_CBS_134524_T
Neogymnomyces_demonbreunii_CBS_427_70_T
Malbranchea_californiensis_UAMH_1889_T
Malbranchea_irregularis_FMR_19016_T
Malbranchea_irregularis_FMR_19030
Malbranchea_thaxteri_UAMH_3912_CBS_248_58T
FMR_20151
Malbranchea_sp_IHEM_28255
Malbranchea_filamentosa_UAMH_4097_T
Malbranchea_stricta_FMR_17680_T
Malbranchea_setosa_CBS_198_92_T
Malbranchea_flava_IFM_41299_T
Malbranchea_dendritica_CBS_131_77_T
Malbranchea_flocciformis_IFM_41298_T
FMR_20852
Malbranchea_umbrina_UAMH_3952_T
Malbranchea_gymnoascoides_FMR_17681_T
Malbranchea_ostraviensis_CCF_4241_T
FMR_20173
Malbranchea_phuphaphetensis_CV00115_T
Malbranchea_aurantiaca_CBS_127_77_T
Malbranchea_circinata_CBS_129_77_T
Malbranchea_conjugata_CBS_247_58_T
Malbranchea_conjugata_UAMH_3874
Malbranchea_chrysosporioidea_IFM_41294_T
Malbranchea_chinensis_CGMCC_3_19572_T
Malbranchea_cavernosa_URM_8445_T
Malbranchea_sinuata_FMR_18266_T
Malbranchea_thaxteri_CBS_319_61
FMR_20150
FMR_21121
FMR_19564
Malbranchea_albolutea_UAMH_2846_CBS_125_77_T
Malbranchea_zuffiana_CBS_127971
Malbranchea_zuffiana_CBS_219_58_T
FMR_20086
Malbranchea_reticulata_UAMH_2006
Malbranchea_reticulata_FMR_18696
Malbranchea_compacta_CBS_200_64_T
Malbranchea_echinulata_FMR_17906_T
Malbranchea_pulchella_IFM_41308_T
Malbranchea_fulva_CBS_135_77_T
Malbranchea_pseudauxarthron_IFO_31701_T
Malbranchea_guangxiensis_CGMCC_3_19634_T
Malbranchea_guangxiensis_LC_12465
Malbranchea_kuehnii_CBS_539_72_T
Malbranchea_pseudoreticulata_UAMH_3117_T
Malbranchea_concentrica_FMR_4054_T
Malbranchea_chlamydospora_RV_24809_T
Malbranchea_chlamydospora_AC0702
Malbranchea_multiseptata_CBS_146931_T
FMR_19403
Malbranchea_longispora_FMR_12768_T
Malbrancheaceae
ITS-LSU-TUB
(1597 bp)
0.10
PTP ITS
ASAP
ASAP ITS

Figure S5 Speciation analysis of *Malbranchea*. The tree represents the bPTP analyses of the concatenated ITS-LSU-*tub*2 alignment of *Malbranchea*. Blue lines indicate speciation events and red lines comprise species. The topology obtained through the other SAP and bPTP analyses is represented on the side. Incongruent topologies are highlighted in red.

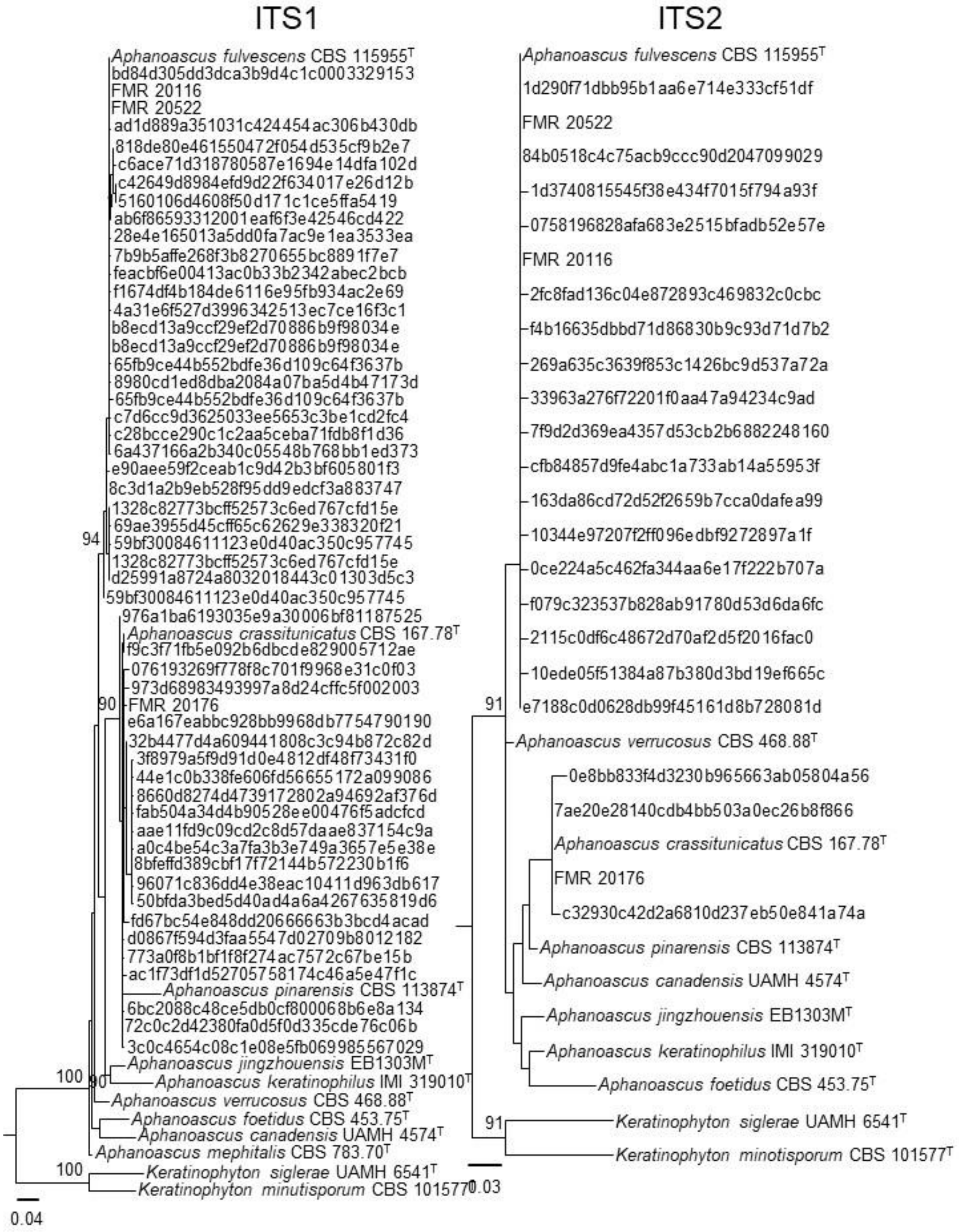

Figure S6 Maximum likelihood tree representing the individual ITS1 and ITS2 alignments of *Aphanoascus*. Sequence codes represent unique identifiers in GlobalFungi.

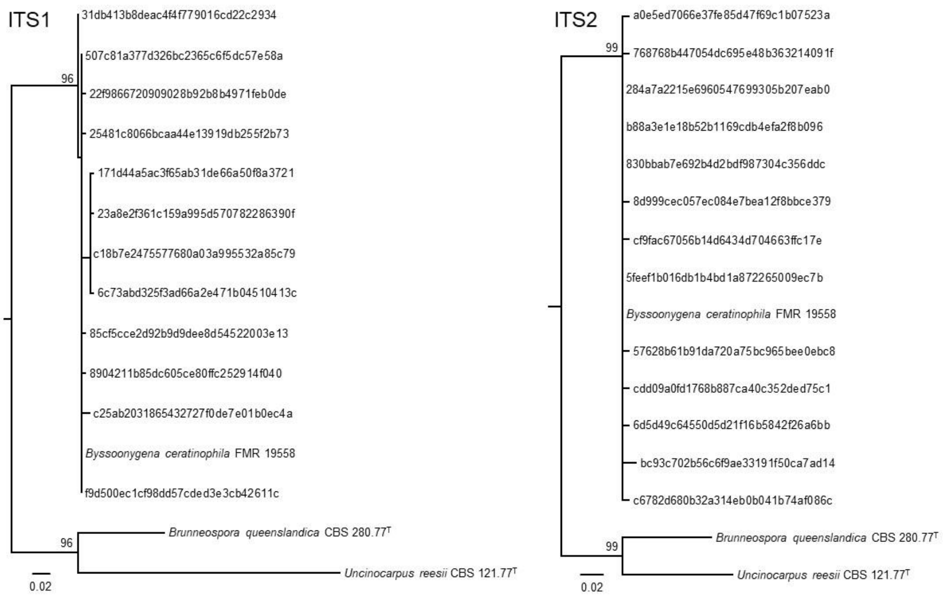

Figure S7 Maximum likelihood tree representing the individual ITS1 and ITS2 alignments of *Byssoonygena.* Sequence codes represent unique identifiers in GlobalFungi.

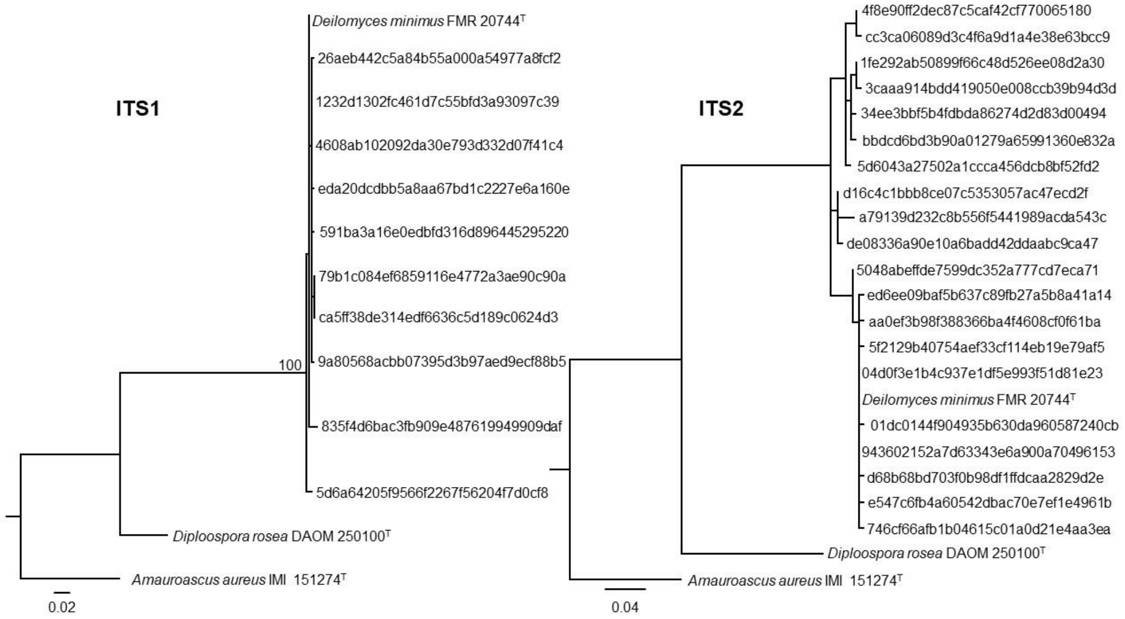

Figure S8 Maximum likelihood tree representing the individual ITS1 and ITS2 alignments of *Deilomyces*. Sequence codes represent unique identifiers in GlobalFungi.

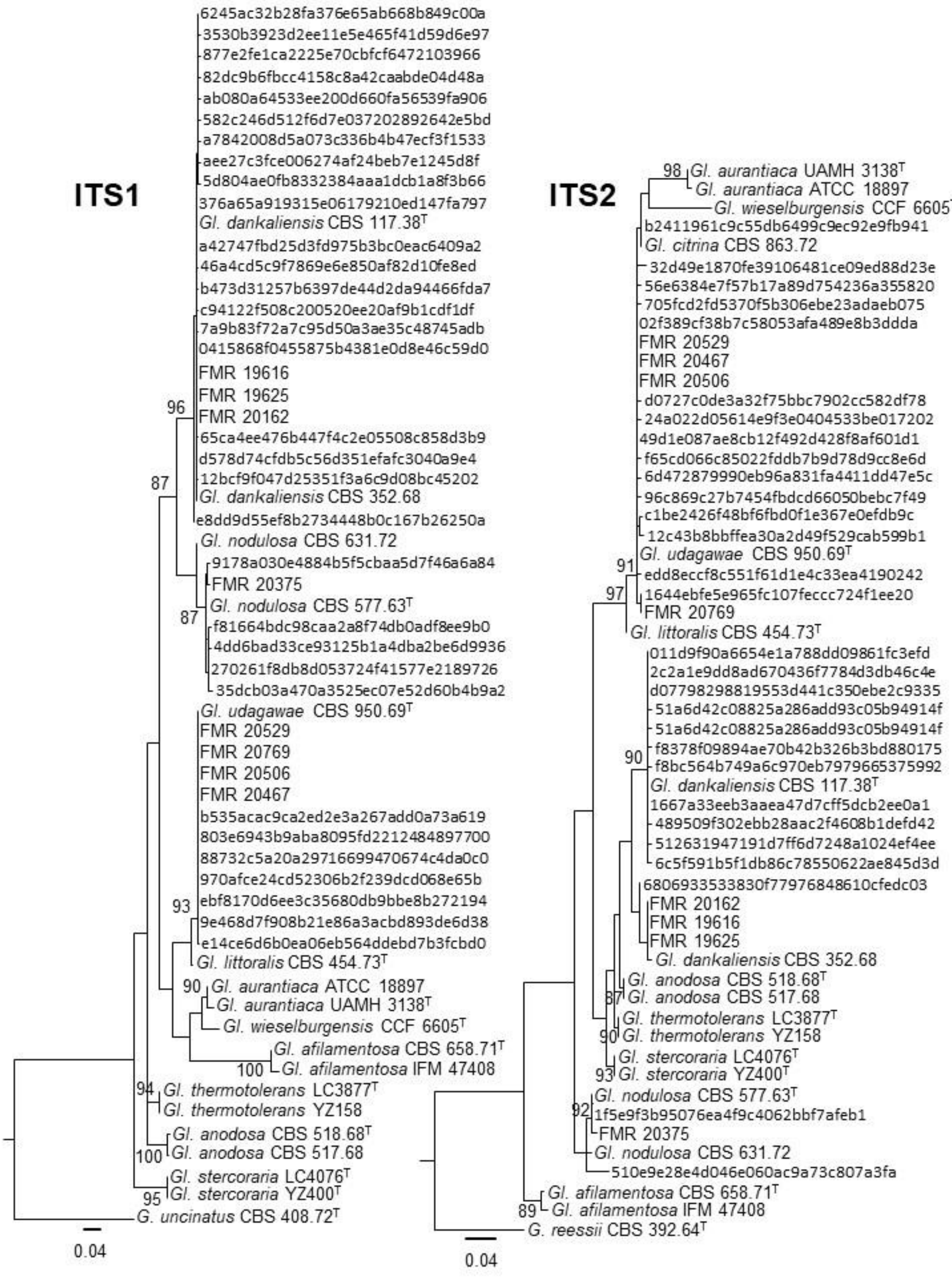

Figure S9 Maximum likelihood tree representing the individual ITS1 and ITS2 alignments of *Gymnascella*. Sequence codes represent unique identifiers in GlobalFungi.

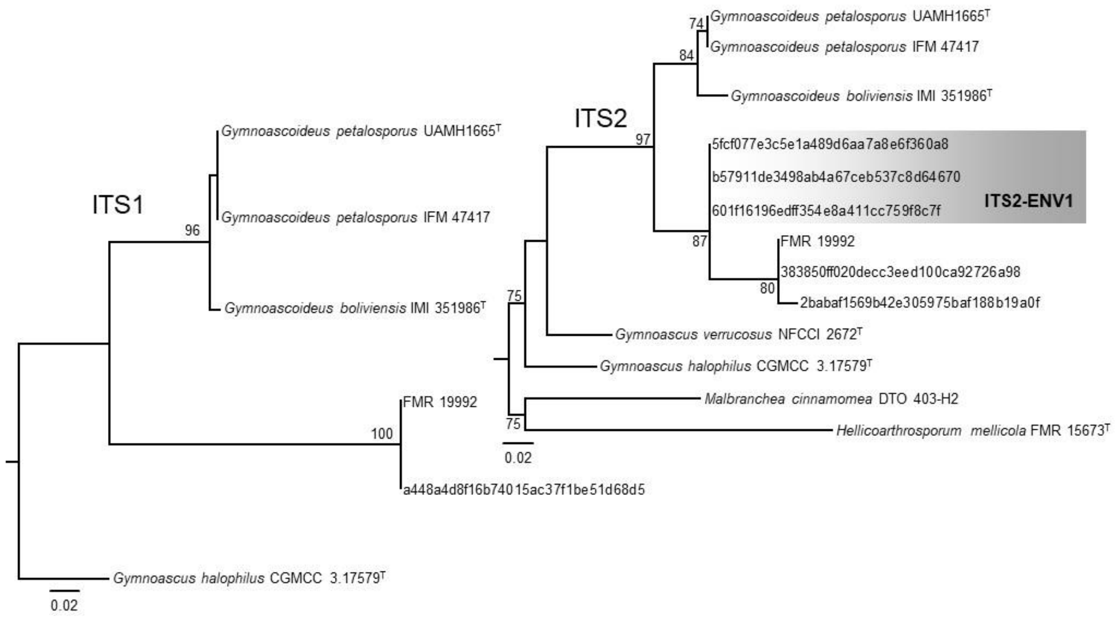

Figure S10 Maximum likelihood tree representing the individual ITS1 and ITS2 alignments of *Gymnascoideus*. Sequence codes represent unique identifiers in GlobalFungi.

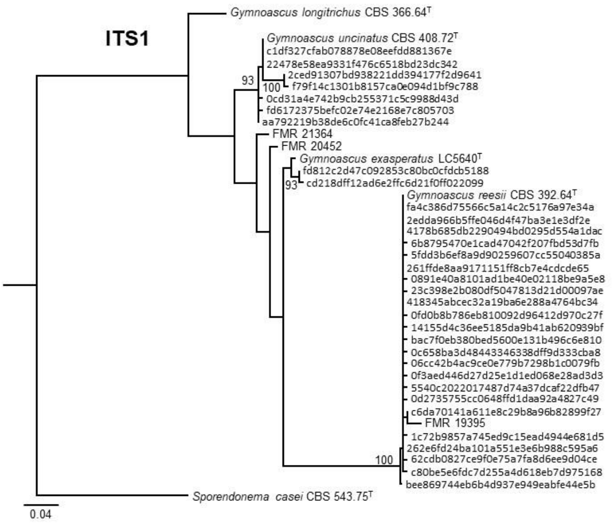

Figure S11 Maximum likelihood tree representing the individual ITS1 alignment of *Gymnoascus*. Sequence codes represent unique identifiers in GlobalFungi.

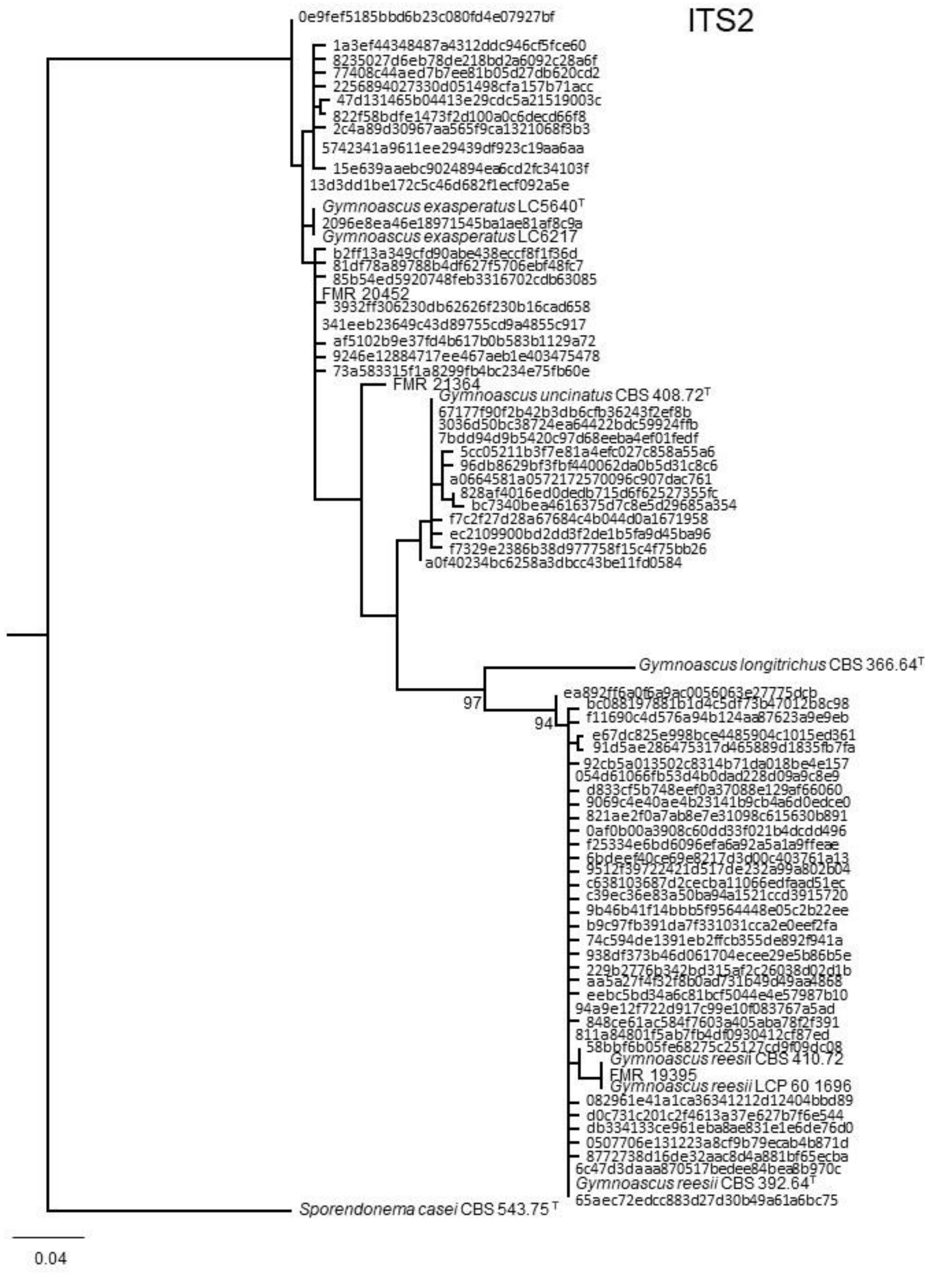

Figure S12 Maximum likelihood tree representing the individual ITS2 alignment of *Gymnoascus*. Sequence codes represent unique identifiers in GlobalFungi.

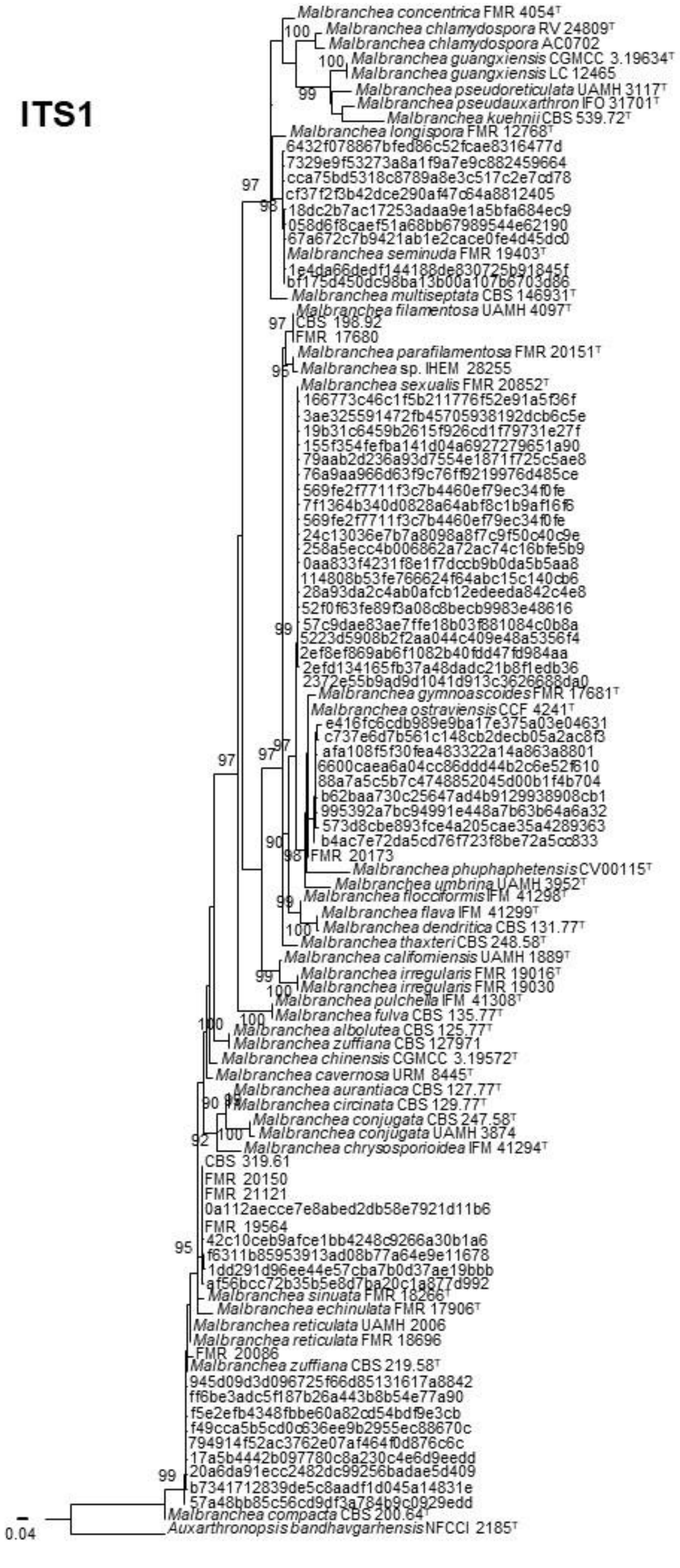

Figure S13 Maximum likelihood tree representing the individual ITS1 alignment of *Malbranchea*. Sequence codes represent unique identifiers in GlobalFungi.

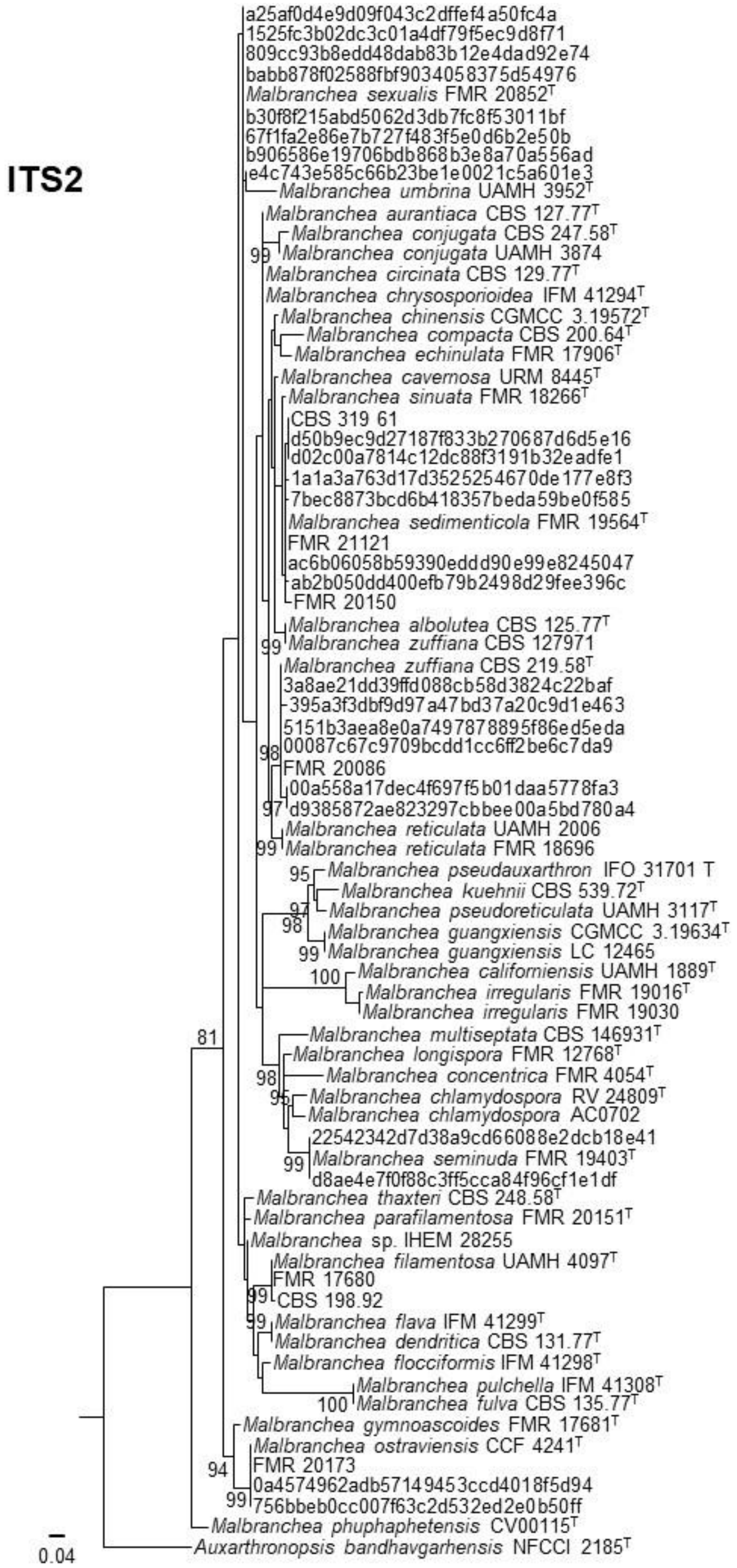

Figure S14 Maximum likelihood tree representing the individual ITS2 alignment of *Malbranchea*. Sequence codes represent unique identifiers in GlobalFungi.

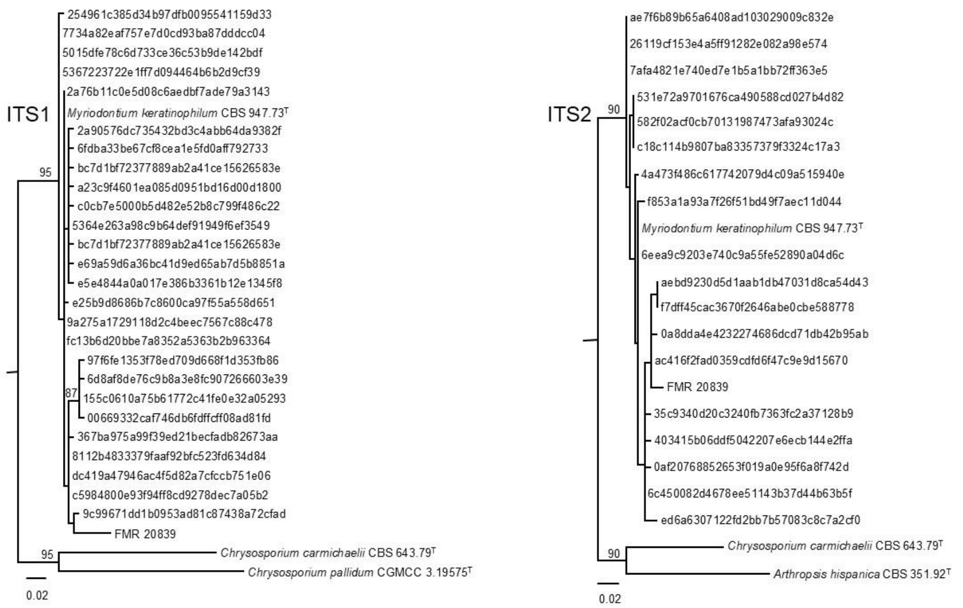

Figure S15 Maximum likelihood tree representing the individual ITS1 and ITS2 alignments of *Myriodontium*. Sequence codes represent unique identifiers in GlobalFungi.

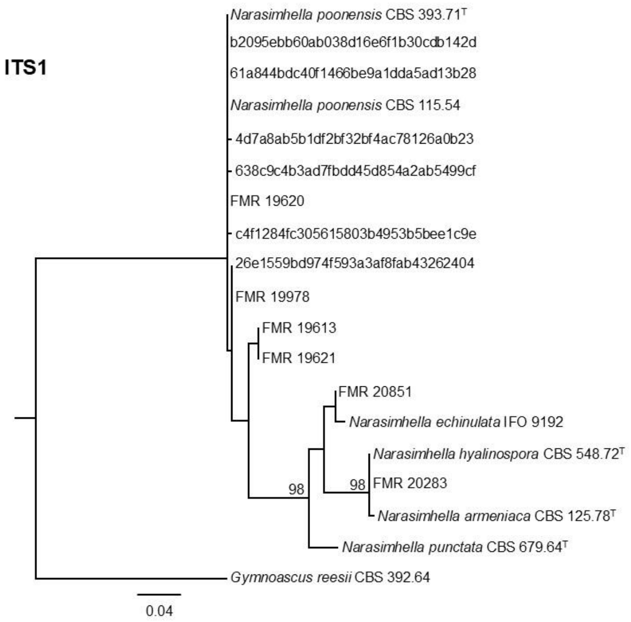

Figure S16 Maximum likelihood tree representing the individual ITS1 alignment of *Narasimhella*. Sequence codes represent unique identifiers in GlobalFungi.

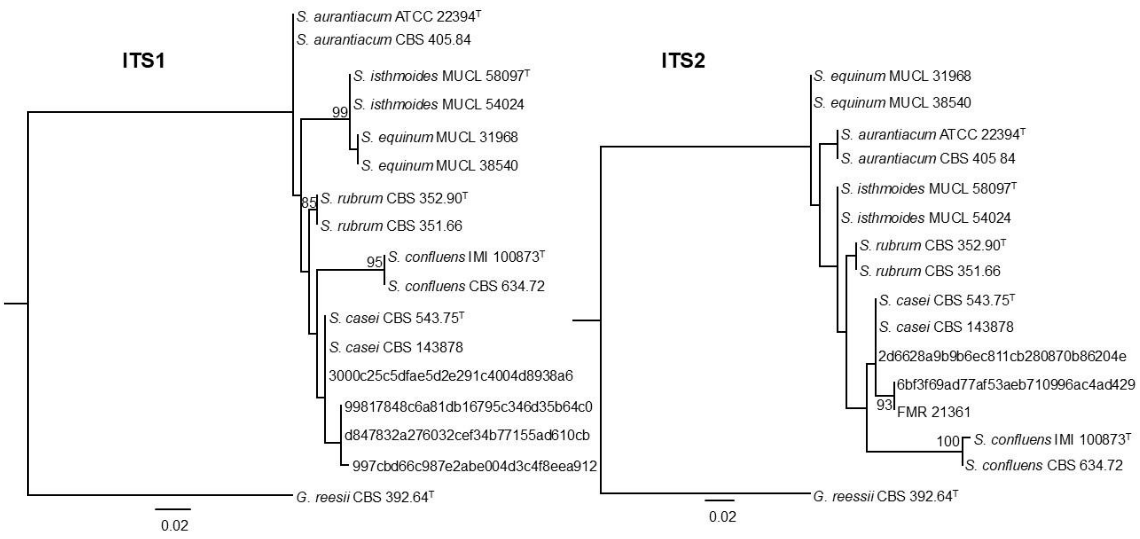

Figure S17 Maximum likelihood tree representing the individual ITS1 and ITS2 alignments of *Sporendonema*. Sequence codes represent unique identifiers in GlobalFungi.

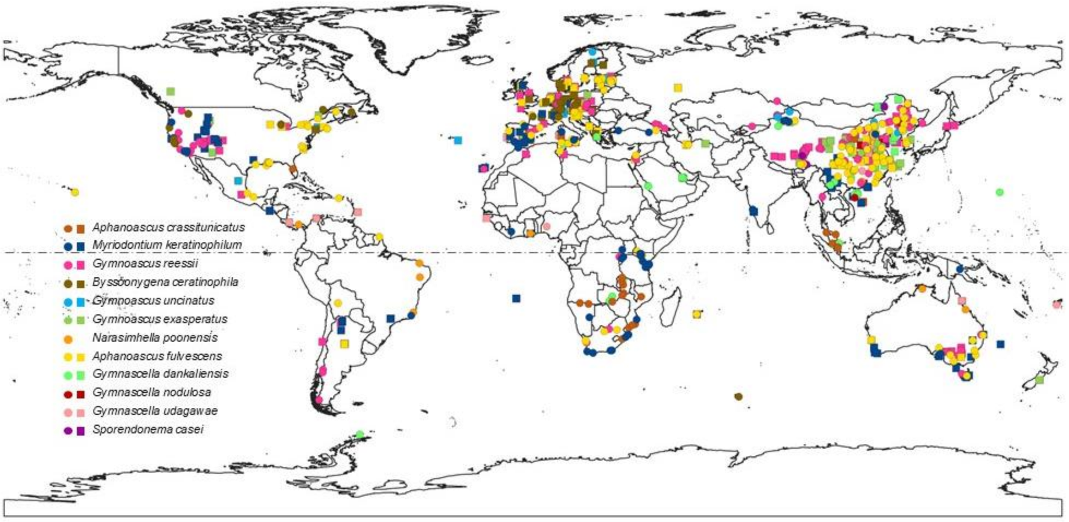

Figure S18 Biogeographical distribution from GlobalFungi of the strains in *Aphanoascus*, *Byssoonygena*, *Gymnascella*, *Gymnoascus*, *Myriodontium*, *Narasimhella* and *Sporendonema*.

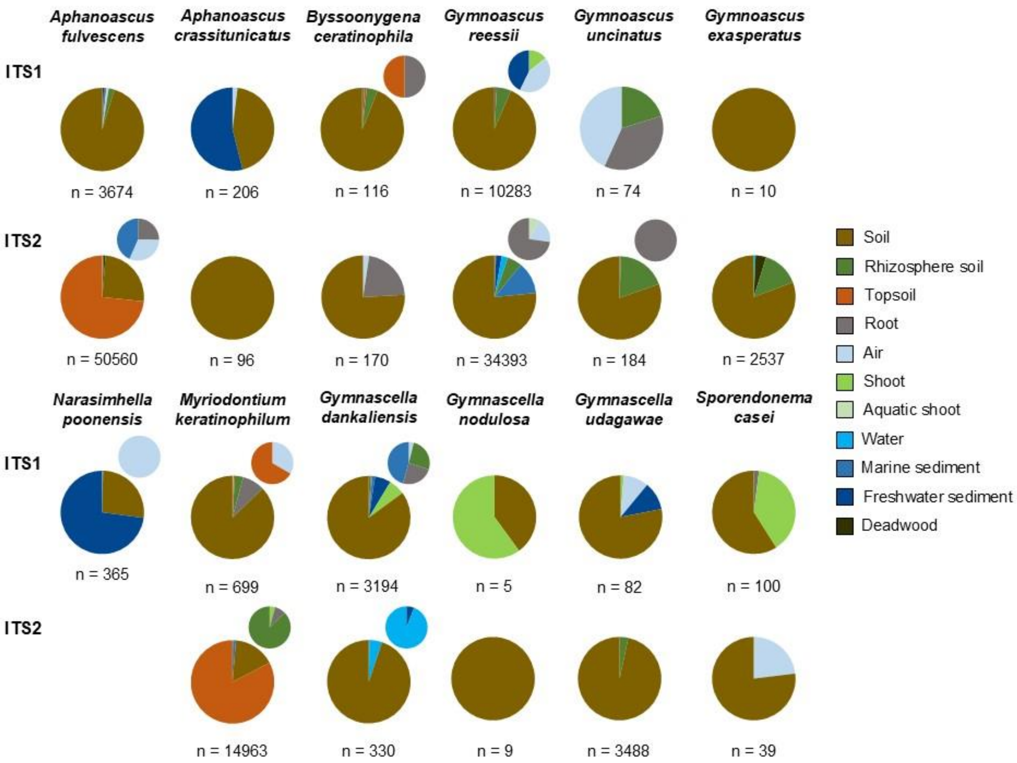

Figure S19 Substrate distribution from GlobalFungi of the strains in *Gymnoascaceae*.
